# Transgenic mouse models of sodium and potassium channelopathies in epilepsy: insights into disease mechanisms and therapeutics

**DOI:** 10.1042/BSR20253356

**Published:** 2025-10-28

**Authors:** Michael F. Hammer

**Affiliations:** BIO5 Institute and Department of Neurology, University of Arizona Tucson, AZ, 85721, U.S.A.

**Keywords:** gain-of-function, injury cascade, loss-of-function, neuronal hyperexcitability, targeted therapies, voltage-gated ion channel

## Abstract

Brain-expressed voltage-gated sodium (Nav) and potassium (Kv) channels are essential for maintaining the balance of neuronal excitability, each having opposing effects on membrane potential and neuronal firing. Genetic alterations in these channels can disrupt this balance, leading to epilepsy and/or developmental impairments through gain-of-function (GoF) or loss-of-function (LoF) mechanisms. This review catalogs 48 transgenic mouse models involving sodium channels (SCN1A, SCN2A, SCN3A, SCN8A) and potassium channels (KCNQ2, KCNQ3, KCNT1, KCNA1, KCNB1, KCND2), detailing the effects of genetic alterations in terms of channel function, affected cell types, and phenotypic manifestations. Mechanistic insights from these models reveal that initial channel dysfunction triggers cascading pathological processes including glutamate excitotoxicity, oxidative stress, gliosis, neuroinflammation, and blood–brain barrier disruption. Therapeutic approaches include antisense oligonucleotides to enhance functional allele expression or reduce pathogenic channel expression, viral-mediated gene therapy, gene editing, and small molecule modulators that target persistent sodium currents or that stabilize channel inactivation. The timing of intervention appears to be critical, with early treatment showing greater efficacy in preventing pathological cascades. Strain-specific background effects and compensatory ion channel expression affect phenotypic severity and treatment response, complicating translation of model results. Importantly, transgenic models offer opportunities to better understand mechanisms underlying comorbidities commonly suffered by patients, including behavioral disorders, motor impairments, and sleep disturbances. The integration of these findings suggests that effective treatment strategies may require combinations of channel-directed therapies and interventions targeting downstream pathological processes, particularly for established disease. This comprehensive examination of channelopathy models provides a framework for developing transformative therapeutics for genetic epilepsies.

## Introduction

### Voltage-gated sodium and potassium channels: Contrasting structure and function

**Figure 1:**
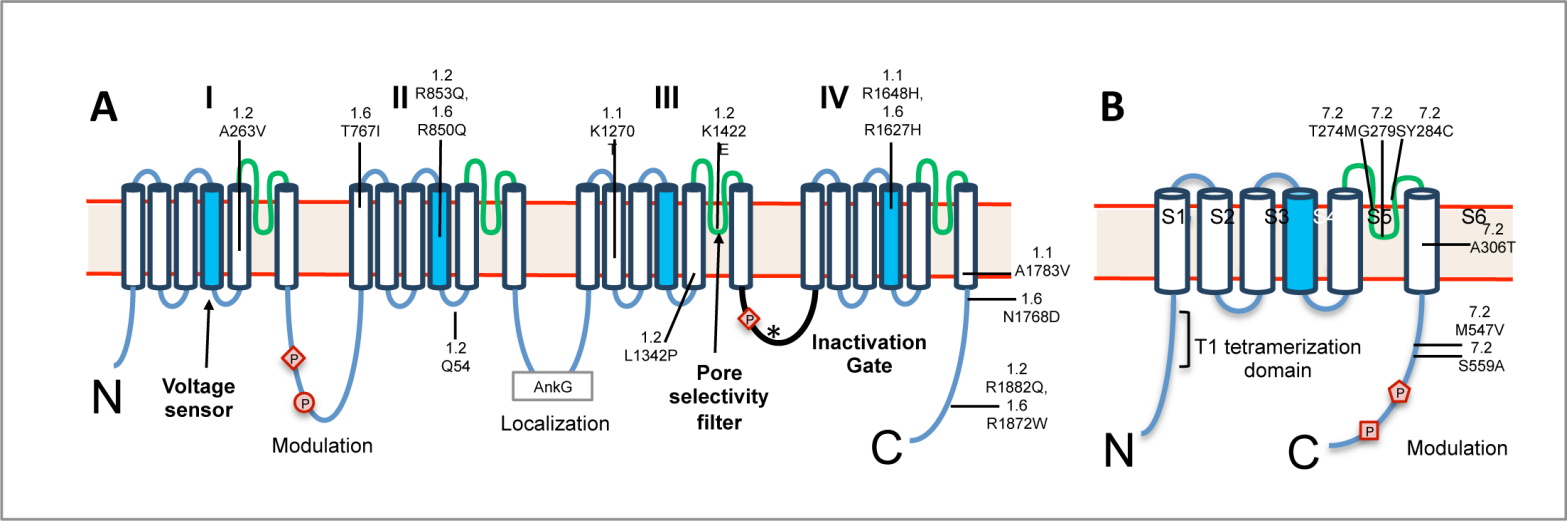
Basic Nav and Kv channel structure. **A.** Diagram of the main configurations and key features of the Nav α‐subunit. The α‐subunit is a composed of a continuous peptide that begins with the N-terminus (**N**) and ends with the C-terminus (**C**) and that is arranged into four domains, designated DI–DIV. Each domain contains six transmembrane segments (S1-6) with S4 acting as the voltage sensor (blue cylinder) and S5 and S6 forming the selectivity filter of the pore (green ribbon), ensuring that only Na ^+^ ions can pass through. The intracellular linker between domains III and IV (highlighted in black) functions as the inactivation gate. Upon depolarization (Figure 2), activation of the domain IV voltage sensor initiates cytoplasmic DIII-DIV linker movement and quickly repositions the IFM (isoleucine-phenylalanine-methionine) motif (asterisk) into the inner mouth of the pore, blocking ion conduction. Examples of regulation of channel function via phosphorylation include protein kinase A (PKA) phosphorylation (circle) at Ser573 in the domain I-II linker of Na_V_1.1 and Na_V_1.2 (see text), protein kinase C (PKC) phosphorylation (diamond) at Ser1506 in the domain III-IV linker, and p38 MAPK phosphorylation at Ser553 in the domain I-II linker in Na_V_1.6 creating a Pro-Gly-Ser-Pro motif that serves as a binding site for the Nedd4 ubiquitin ligase (see text). The ankyrin G-binding motif within intracellular loop 2 (AnkG in black box) plays a role in channel targeting to axonal compartments. **B**. Schematic diagram of structural elements present in most Kv α‐subunits. The α‐subunit consists of six transmembrane segments (**S1-S6**), with S1-S4 forming the voltage sensor and S5-S6 lining the pore. Kv channels are formed by four alpha subunits that assemble to form a functional channel (tetramerization). A region between S5 and S6 contributes to the selectivity filter of the pore, ensuring that only K^+^ ions can pass through. Approximate positions of several variants mentioned in the text are shown with arrows. Examples of regulation via phosphorylation include CDK5 phosphorylation of K_V_2.1 channels at Ser-516, Ser-603, Ser-651, and Ser-800 (square), AMPK phosphorylation at Ser-440 (pentangle), and CaMKII phosphorylation of K_V_4.2 at Ser438, all of which play roles in modulating neuronal excitability.

**Figure 2:**
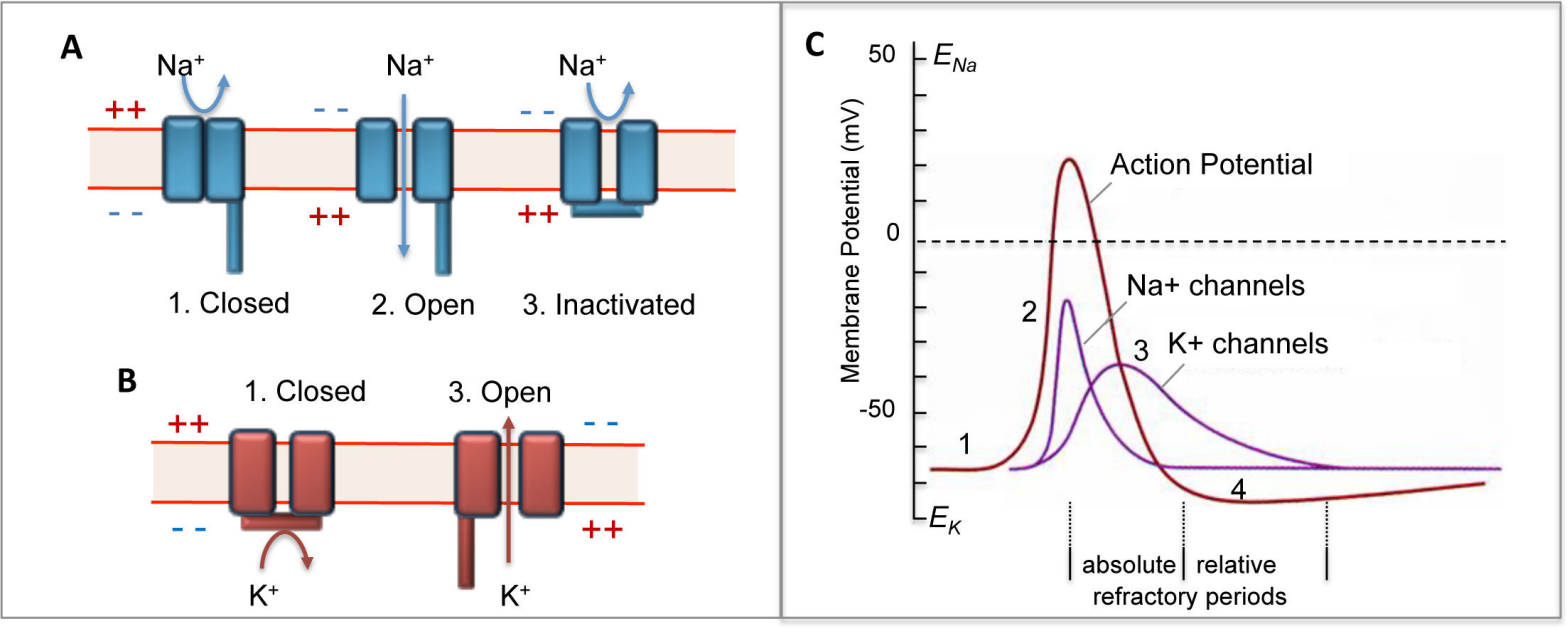
Diagrams of Nav and Kv channels and changes in Na^+^ ion and K^+^ ion conductance during the course of an action potential (AP). **A.** The fundamental gating cycle for sodium channels involves three primary conformational states: closed (deactivated), open (activated), and inactivated. Fast inactivation occurs within milliseconds through the cytoplasmic inactivation gate formed by the III-IV linker (Figure 1) that physically blocks the pore, while slow inactivation involves conformational changes in the selectivity filter. Recovery from inactivation must occur before reopening, creating absolute and relative refractory periods that control AP frequency. **B.** Kv channels share the basic closed → open transition driven by membrane depolarization, where voltage sensors (S4 segments) move in response to electric field changes to open the activation gate. However, gating patterns vary depending on Kv subfamily: Kv7 (KCNQ) channels exhibit minimal inactivation and maintain sustained currents during prolonged depolarization, while Kv1, Kv2, and Kv3 channels include rapid inactivation through N-type (ball-and-chain) or C-type (selectivity filter) mechanisms. Kv4 channels show particularly fast A-type inactivation similar to sodium channels, enabling precise control of dendritic excitability and spike timing. **C**. 1) At rest the activation gates of both channels are closed, and the channels are not inactivated; 2) during the upstroke of the AP, depolarization causes the activation gates of the Na^+^ channel to open. This produces a large increase in the permeability to Na^+^ ions. The Kv channels also begin to open but much more slowly so that most are still closed during the upstroke; 3) during the repolarizing phase, the Nav channel inactivation gate closes and the activation gates of the Kv channels are now fully open. This point corresponds to the absolute refractory period because the Nav channels are inactivated and are not ready to re-open and initiate another AP; 4) during the afterhyperpolarization phase, the Kv channels are still open and the Nav channels are beginning to recover from inactivation. This point corresponds to the relative refractory period [3].

Voltage-gated sodium (Nav) and potassium (Kv) channels have opposing effects on neuronal excitability, creating complex relationships between genetic alterations and epilepsy phenotypes [1,2]. The Nav channel α-subunit contains four transmembrane domains separated by intracellular loops, while the Kv channel α-subunit contains a single domain with six transmembrane segments (S1-S6) that assembles as a tetramer (Figure 1). This structural difference mirrors their distinct functional roles: Nav channels trigger action potentials (AP) through sodium influx, while Kv channels terminate excitation and set resting potential through potassium efflux (Figure 2). Gain-of-function (GoF) variants in Nav channels enhance neuronal firing through premature channel opening, increased persistent current, or delayed inactivation, leading to hyperexcitability and seizures [4,5]. Conversely, loss-of-function (LoF) Nav variants typically reduce excitatory neuron firing and associate with neurodevelopmental disorders [6]. For Kv channels, the relationship is generally reversed: LoF variants increase neuronal excitability through reduced hyperpolarizing currents and cause seizures, while GoF variants can have protective effects [7,8].

### Distinctions within channel types

**Figure 3:**
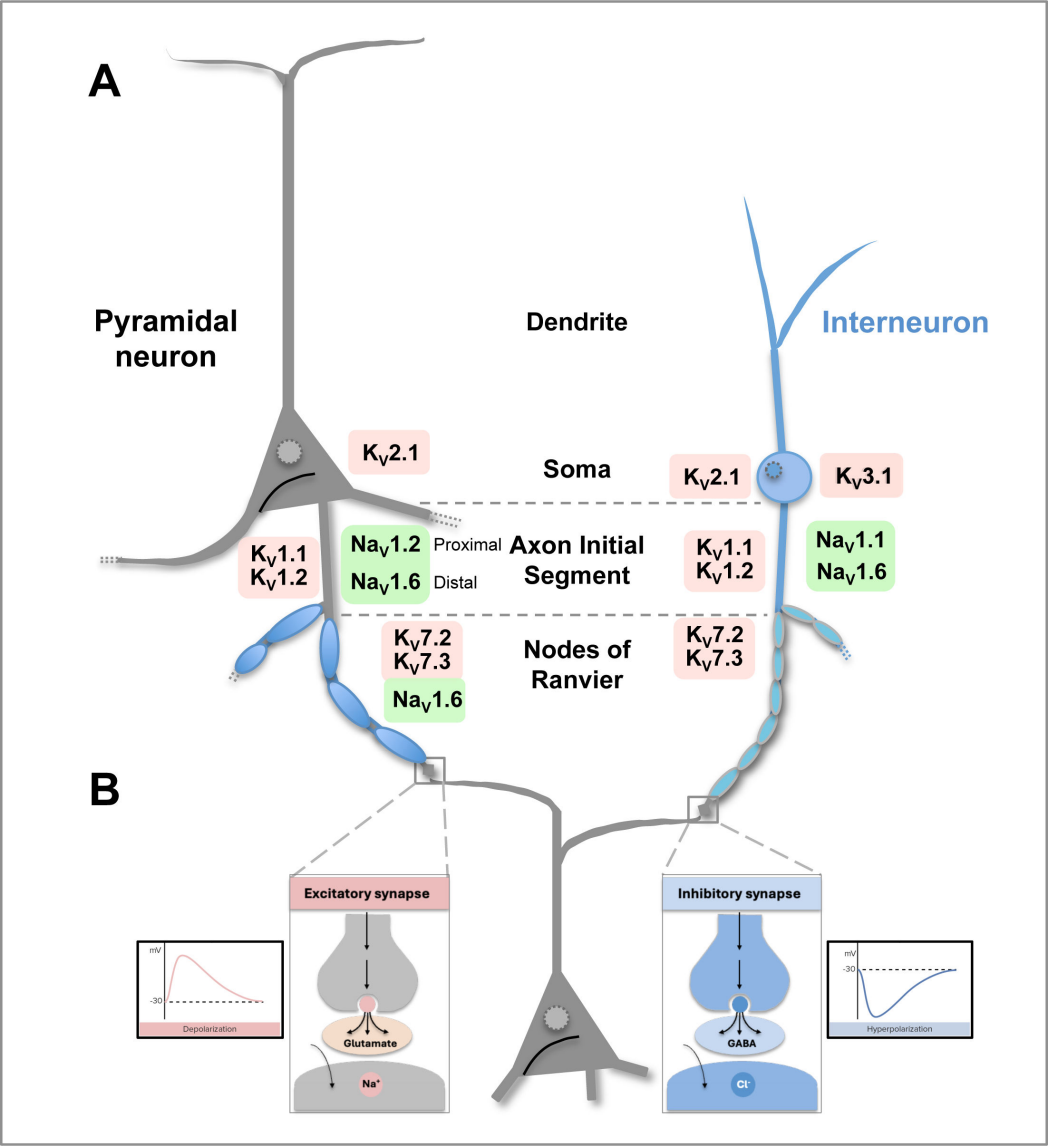
Location and expression of voltage-gated Nav and Kv channels in excitatory and inhibitory neurons. **A**. Schematic illustrating the known expression patterns of ion channels implicated in genetic epilepsy. Gene groups are represented by different colors (after [11]. Na_V_1.1 subunits are expressed predominantly in inhibitory GABAergic neurons and are enriched at the axon initial segment (AIS), playing a role in the initiation and propagation of APs in these cells. Na_V_1.2 channels—expressed predominantly in excitatory neurons—are clustered at the proximal part of the AIS where they play an important role in promoting back propagation to the soma and dendrites. Na_V_1.6 density is higher in the distal part of the AIS where they play a key role in AP initiation. They are also expressed at the nodes of Ranvier (NOR) where they regenerate and amplify APs during saltatory conduction in myelinated axons, enabling rapid and efficient signal transmission over long distances. K_V_1.1 and K_V_1.2 are predominantly localized in the AIS, axon preterminal, and the juxtaparanodal domain adjacent to the NOR, where they help to repolarize and shape APs. Kv2.1 subunit is expressed in both excitatory and inhibitory neurons, and is localized to the soma, proximal dendrites, and AIS. K_V_3.1 is primarily expressed at somatic/axonal membrane, while K_V_7.2 and K_V_7.3 colocalize at the AIS and/or NOR, where they play an integral role in defining excitability. Not shown are the β1-ancillary subunits of Nav channels (Na_V_β1), which modulate channel gating, regulate the level of channel expression, and potentially act as cell adhesion molecules. Na_V_β1 are enriched at AIS and nodes of Ranvier of both excitatory and inhibitory neurons. **B**. Left: excitatory synapse using an excitatory postsynaptic potential (EPSP) and the neurotransmitter glutamate to create a positive depolarization. Right: inhibitory postsynaptic potential (IPSP) using the neurotransmitter GABA, causing hyperpolarization.

In excitatory neurons, Nav1.2, Nav1.6, and Nav1.3 (encoded by SCN2A, SCN8A, and SCN3A, respectively) are differentially distributed: Nav1.2 predominates in the proximal axon initial segment (AIS), while Nav1.6 localizes to the distal AIS and nodes of Ranvier (NOR) [9,10] (Figure 3). During development, Nav1.2 at immature nodes is replaced by Nav1.6 during nodal maturation [12]. Nav1.1 (SCN1A) presents a unique case through its predominant expression in GABAergic interneurons. SCN1A LoF variants primarily affect inhibitory circuits, creating a paradox where LoF mutations produce epilepsy phenotypes similar to GoF variants in excitatory Nav channels, but through reduced inhibition rather than direct hyperexcitation [13,14]. This cell-type specificity explains why genetic variants with seemingly opposite effects can produce similar network-level hyperexcitability [1,15].

Kv channels serve multiple regulatory functions beyond AP repolarization. Kv7 (KCNQ) channels provide sustained ‘M-current’ that maintains resting potential through slow activation kinetics [16], while Kv4 channels generate rapidly inactivating A-type currents crucial for dendritic excitability [17]. Mutations in KCNA1, KCNB1, KCND2, KCNT1, and KCNQ2 typically cause epilepsy through reduced hyperpolarizing function (Table 1).

**Table 1:** Transgenic mouse models of sodium and potassium channelopathies

Gene (Protein)	Mutation	Location in channel	Functional change	Functional effects	Cell types affected	Phenotypes	Citation
Scn1a (Nav1.1)	Conditional KO	Complete gene/channel	Loss	Reduced Na^+^ currents	Parvalbumin + interneurons	Severe seizures, Premature death	[18]
	Exon 1, 26 deletions	Multiple domains (deletion)	Loss	Reduced Na^+^ currents	Inhibitory neurons	Seizures, Early mortality	[18,19]
	R1648H	DIV S4 (voltage sensor)	Loss	Reduced Na^+^ current	GABAergic interneurons	GEFS + phenotype, Temperature-sensitive seizures, Normal lifespan	[20]
	K1270T	DIII S6 (pore region)	Loss	Reduced current	Multiple neuron types	Temperature-sensitive seizures, Behavioral abnormalities	[21]
	R1407X	DIV S1-S2 linker	Loss	Protein truncation	Inhibitory neurons	Severe epilepsy, Early-onset seizures, High mortality	[11]
	A1783V	C-terminus	Loss	Reduced Na^+^ current	Multiple neuron types	Dravet phenotype, Spontaneous seizures, Cognitive deficits, Autistic-like behaviors	[22]
	E1099X	DII-III linker	Loss	Protein truncation	GABAergic interneurons	Severe dravet syndrome, Early-onset seizures, High mortality	[23]
Scn2a (Nav1.2)	Complete KO	Complete gene/channel	Loss	No Nav1.2 expression	Multiple neurons	Limited survival, Developmental impairment	[24,25]
	Excitatory neuron-specific KO	Complete gene/channel	Loss	No Nav1.2 in excitatory neurons	Excitatory neurons	Limited survival, elevated neuronal excitability	[26]
	Q54	DI S4-S5 linker	Gain	Increased persistent current	Hippocampal neurons	Epilepsy, Spontaneous seizures	[27]
	R853Q	DII S4 (voltage sensor)	Mixed	Reduced current, decreased excitability	Cortical neurons	DEE phenotype, Decreased excitability, Cognitive deficits	[28]
	R1882Q	C-terminus	Gain	Enhanced activation	Pyramidal neurons	Early seizures	28]
	L1342P	Diii s5	Gain	Enhanced activation	Cortical neurons	Early-onset seizures, hyperexcitability	[29]
	K1422E	DIV S1 segment	Mixed	Altered ion permeability	Excitatory neurons	Rare spontaneous seizures, Interictal EEG abnormalities, Altered social behavior	[30]
	A263V	DI S5 segment	Gain	Enhanced activation	Multiple neurons	Severe epilepsy, altered dendritic excitability	[31,32]
Scn8a (Nav1.6)	N1768D	DIV S4 (voltage sensor)	Gain	Enhanced activation	Excitatory neurons	Severe seizures, Premature death	[4]
	R1872W	C-terminus	Gain	Enhanced activation	Multiple neurons	Early (neonatal) seizures, Premature death	[33,34]
	T767I	DII S1 segment	Gain	Enhanced activation	Pyramidal neurons	Epilepsy, Spontaneous seizures, Premature death	[35]
	R1627H	DIV S4 (voltage sensor)	Gain	Increased current	Multiple neurons	Seizures, Heightened neuronal excitability	[36]
	R1620L	DIV S4 (voltage sensor)	Mixed	Enhanced activation with LoF effects	Excitatory neurons, reduced function	Epilepsy, Autistic-like behavior, Spontaneous seizures	[37]
	medJ*	Multiple domains	Loss (90%)	Severely reduced function, slowed nerve conduction velocity	Pyramidal neurons	Severe dystonia	[38]
	A1071T (med-jo)	DII-DIII linker	Loss	Reduced current	Multiple neurons	Mild phenotype, Absence seizures, Ataxia	[39]
	??irl/+	C-terminus (deletion)	Loss	Altered function	Multiple neurons	Increased seizure susceptibility, Infrequent spontaneous seizures	[40]
	??vir//+	C-terminus (deletion)	Loss	Altered function	Multiple neurons	Increased seizure susceptibility, Infrequent spontaneous seizures	[41]
	F210S	DI S5 segment	Loss	Reduced function	Multiple neurons	Mild phenotype	[39,42]
	V929F (8 j)	DII-DIII linker	Loss	Reduced current	Cerebellar neurons	Ataxia, Absence seizures	[41]
	Med (null)	Complete channel	Loss	No Nav1.6 expression	Multiple neurons	Severe movement disorder, perinatal lethality	[43]
Scn3a (Nav1.3)	Scn3aHyp	Multiple domains affected	Gain	Enhanced activation	Multiple neurons	Seizure susceptibility, Increased neuronal excitability	[44]
Kcnq2 (Kv7.2)	Complete KO	Complete gene/channel	Loss	No M-current	Hippocampal neurons	Severe seizures	[45]
	Conditional KO	Complete gene/channel	Loss	Cell-specific loss of M-current	Selected neurons	Variable severity depending on cell type	[46]
	Y284C (conditional)	S4-S5 linker	Loss	Reduced current	Multiple neurons	Epilepsy, Behavioral hyperactivity, Cognitive deficits	[47]
	T274m (KI)	S4 segment (voltage sensor)	Loss	Reduced current	Hippocampal neurons	Early generalized seizures, Reduced lifespan, Deficits in spatial learning	[48]
	A306t (KI)	S5 segment	Loss	Reduced function	Multiple neurons	Neonatal seizures, Neurodevelopmental impairment	[49]
	S559a (KI)	C-terminus	Loss	Altered function	Cortical neurons	Mild phenotype, Altered seizure susceptibility	[50]
	G279S (cond KI)	S4 segment (voltage sensor)	Loss	Reduced current	Selected neurons	Variable effects, Reduced seizure burden	[51]
	M547V (Cond KI)	C-terminus	Loss	Reduced current	Selected neurons	Mixed phenotype, Spontaneous seizures, Memory loss	[52]
KCNA1 (Kv1.1)	Complete KO	Complete gene/channel	Loss	No Kv1.1 expression	Multiple neurons	Severe temporal lobe seizures, Premature death	[53]
	Neuron-specific KO	Complete gene/channel	Loss	No Kv1.1 in neurons	Selected neurons	Epilepsy, Premature death, Cardiorespiratory dysregulation	[54]
	V408A	S6 segment (pore region)	Loss	Reduced K^+^ current	Multiple neuron types	Episodic ataxia type 1, Stress-induced seizures	[55,56]
KCNB1 (Kv2.1)	Complete KO	Complete gene/channel	Loss	No Kv2.1 expression	Multiple neurons	Spontaneous seizures, Neuronal hyperexcitability, Spatial learning deficits, Hyperactivity	[57]
	G379R	S5-S6 loop (pore forming)	Loss	Reduced K^+^ current	Multiple neuron types	DEE, Spontaneous seizures, Motor impairments, Cognitive deficits	[58,59]
	R306C	S3 segment	Loss	Reduced K^+^ current	Multiple neuron types	Pronounced hyperactivity, Higher seizure susceptibility, Spike wave discharges	[60]
	T374I	S4 segment (voltage sensor)	Loss	Reduced K^+^ current	Multiple neuron types	DEE	[61]
KCND2 (Kv4.2)	Heterozygous KO	Complete gene/channel	Loss	Reduced Kv4.2 expression	Multiple neuron types	Altered neuronal excitability, Dendritic spine abnormalities	[62]
KCNT1 (Slack)	Complete KO	Complete gene/channel	Loss	No slack expression	Multiple neurons	Lower seizure susceptibility, Motor deficits, Learning deficits	[63]
	R455H	S4-S5 linker	Gain	Increased K^+^ and Na^+^ currents	Excitatory and inhibitory neurons	Spontaneous seizures, Interictal discharges, Enhanced seizure susceptibility	[64]
	Y777H	C-terminus	Gain	Large K^+^ conductance	Inhibitory interneurons	Spontaneous tonic and tonic-clonic seizures (homozygotes), Disinhibition of neuronal circuits	[65]
	L437F	S4-S5 linker	Gain	Large K^+^ conductance	Inhibitory interneurons	Increased seizure susceptibility	[66]

### Channel regulation and complexity

Channel function extends beyond the primary α-subunit through auxiliary subunits and dynamic post-translational modifications. Nav channels associate with β-subunits that modulate gating and trafficking, while Kv channels form complexes with diverse auxiliary subunits (KChIP, DPPX, KCNE) that dramatically expand their functional properties [17,67]. Activity-dependent phosphorylation provides real-time regulation, with different kinases creating homeostatic mechanisms that link cellular activity and energy status to channel function [68,69] (Figure 1). This regulatory complexity creates multiple potential therapeutic targets beyond primary channel defects.

### Implications for transgenic mouse models

Understanding these complex channel interactions and cell-type specific effects is crucial for interpreting transgenic mouse models of epilepsy. The opposing functions of Nav and Kv channels, combined with their diverse expression patterns and regulatory mechanisms, create multiple therapeutic targets while requiring careful consideration of network-level consequences [70]. Transgenic mouse models provide essential tools for dissecting these mechanisms and developing targeted therapies for the genetic epilepsies.

## Transgenic mouse models of epilepsy: roles of LoF and GoF variants

Mouse models involving voltage-gated ion channels have been instrumental in understanding the pathophysiology of genetic epilepsy syndromes. Table 1 lists 48 mouse models involving Nav (SCN1A, SCN2A, SCN3A, SCN8A) and Kv (KCNQ2, KCNQ3, KCNT1, KCNA1, KCNB1, KCND2) channels.

### Sodium channel models

#### **Scn1a (Na_V_1.1**)

Clinical presentation of patients with pathogenic variants in SCN1A ranges from febrile seizures alone and Genetic Epilepsy with Febrile Seizures Plus (GEFS+) to more severe phenotypes like Dravet syndrome (DS). The fundamental mechanism across SCN1A models stems from LoF effects that primarily affect GABAergic inhibitory interneurons, particularly parvalbumin-positive cells [13]. While patients with SCN1A GoF variants and symptoms that resemble SCN2A and SCN8A DEEs are known [20], there are as of yet no studies of transgenic mice with GoF variants [21]. The severity of the phenotype correlates with the degree of channel dysfunction, creating a spectrum of presentations that closely mirror human disease. At the milder end, the R1648H variant in the DIV S4 (voltage sensor) (Table 1), identified in GEFS+patients, causes partial LoF through impaired channel inactivation, resulting in temperature-sensitive seizures yet normal lifespan [22]. The K1270T variant in DIII S6 (pore region) (Figure 1A) represents an intermediate phenotype, demonstrating both reduced channel expression and altered gating properties, leading to temperature-sensitive seizures with additional behavioral abnormalities [23]. The A1783V variant in the C-terminus underlies a more severe phenotype characteristic of DS, exhibiting dramatically reduced sodium currents in inhibitory neurons that result in spontaneous seizures, cognitive deficits, and autistic-like behaviors [71]. At the most severe end of the spectrum, protein-truncating mutations like R1407X and E1099X cause complete LoF in affected neurons, closely mimicking severe human DS with early-onset seizures and high mortality [13,30].

#### **Scn2a (Na_V_1.2**)

Pathogenic variants in SCN2A are implicated in a spectrum of neurodevelopmental disorders including developmental and epileptic encephalopathies (DEE), familial neonatal-infantile seizures (BFNIS), episodic ataxia, autism spectrum disorder (ASD), and intellectual disability (ID) with and without seizures [26]. SCN2A variants with GoF properties are closely associated with unprovoked seizures and epilepsy, whereas those with LoF effects (missense and protein-truncating variants) are typically associated with ASD and ID [24,72]. Mouse models with GoF or LoF variants generally mimic this spectrum with some interesting caveats. Homozygous knockout (null) of Scn2a in mice is perinatal lethal, whereas heterozygous knockout (Scn2a^+/-^) results in a 40–50% reduction in Nav1.2 expression that only results in mild behavior abnormalities—although absence-like seizures were reported in adult male Scn2a^+/−^ mice [73]. Interestingly, sex-specific differences have recently been reported in this model, where females show a milder phenotype including altered sociability and decision-making behaviors, supporting the male-biased prevalence observed in human ASD [27]. A more profound LoF phenotype, generated using a targeted gene-trap knockout (gtKO) strategy [29], yields an ~75% reduction in Nav1.2 expression compared with wildtype (WT) and produces viable homozygous mice (Scn2a^gtKO/gtKO^) that can survive to adulthood (Table 1). Scn2a^gtKO/gtKO^ mice exhibit a host of behavioral impairments whereby nesting and mating are profoundly disrupted [29,72]. Importantly, this LoF model demonstrates enhanced intrinsic excitability of principal neurons in brain regions known to be involved in Scn2a-related seizures, which might help to explain why 20–30% of patients with Nav1.2 deficiency develop seizures. This counterintuitive finding occurs because Na_V_1.2 loss disrupts the normal balance of excitatory and inhibitory currents at the AIS, ultimately leading to enhanced firing despite reduced sodium conductance (see Cell-Type Specificity section for detailed mechanism) [72].

The GoF spectrum is exemplified by several key models. Introduction of the GAL879-881QQQ mutation in DII S4-S5 linker by mutagenesis (Table 1) resulted in the development of the Q54 transgenic model, which provided the first evidence that increased persistent sodium current in hippocampal neurons leads to enhanced excitability and spontaneous seizures [31]. The GoF variants R1882Q (C-terminus) and L1342P (DIII S5 pore domain) (Figure 1A) enhance channel activation in pyramidal and cortical neurons, respectively, resulting in early-onset seizures [4,28]. The A263V variant in the DI S5 segment shows enhanced activation yet produces a more complex phenotype affecting multiple neuron types [74]. A particularly informative model harboring the K1422E variant in the DIV S1 segment demonstrates more nuanced effects on Nav1.2 channel function that exhibits rare spontaneous seizures, interictal EEG abnormalities, and altered social behavior [24]. The R853Q variant in the DII S4 segment (voltage sensor), associated with DEE, exemplifies how mixed GoF/LoF with more complex biophysical effects affects channel expression and impaired gating, which leads to decreased neuronal excitability and cognitive deficits [75].

#### **Scn8a (Na_V_1.6**)

Patients with pathogenic variants in SCN8A present with a spectrum of phenotypes, including mild-to-severe DEE, pharmacoresistant epilepsy with multiple seizure types, self-limited familial infantile epilepsy (SeLFIE), and neurodevelopmental delays with or without generalized epilepsy [33]. Hypotonia and movement disorders, including dystonia, ataxia, and choreoathetosis, are common in some phenotypes. Similar to SCN2A, models of SCN8A-related disorders include both GoF and LoF mechanisms. The GoF variants demonstrate a consistent pattern of enhanced channel activation and increased persistent current, although with varying severity and specific manifestations. The N1768D model (Figure 1A), initially engineered using TALEN technology [34], exemplifies severe GoF effects leading to pronounced seizures and premature death in C57BL6/J mice (Table 1) [5]. A conditional mouse model was constructed in which expression of the severe GoF variant R1872W (C-terminus), highly recurrent in human patients, is dependent upon Cre recombinase [35,76]. Both global and neural activation of the mutation result in early (neonatal) convulsive seizures and death, while restriction of expression to excitatory neurons results in later (juvenile) seizures and lethality. Activation of the mutation in adult mice also is sufficient to generate seizures and death.

The human-derived T767I knock-in mutation in DII S1 segment (Figure 1A) causes enhanced channel activation in pyramidal neurons, resulting in spontaneous seizures and premature death [37]. The R1627H mutation in the DIV S4 voltage sensor increases channel activity and persistent current, thereby causing severe seizures through heightened neuronal excitability [40]. The constitutive knock-in of R1620L (Table 1), while mapping to a similar voltage sensor region, has mixed GoF and LoF effects that lead to complex phenotypes, including autistic-like behavior and spontaneous seizures [77]. Two models with overlapping in-frame deletions in the DIV voltage sensor (ΔIRL/+ and ΔVIR/+) demonstrate intermediate effects with increased seizure susceptibility yet only infrequent spontaneous seizures [42]. In contrast, LoF variants present distinct phenotypes, often manifesting as movement disorders rather than epilepsy, as demonstrated by the spontaneous med (null) mutation [78]. The V929F (8 j) (DII pore region) and A1071T (med-jo) (DIII S4-S5 loop) variants lead to absence seizures and ataxia, primarily affecting cerebellar neurons [44].

#### **Scn3a (NaV1.3**)

Pathogenic variants in the SCN3A gene are associated with varying degrees of epilepsy, developmental delay, intellectual disability, speech impairments, and occasional brain malformations such as polymicrogyria [79]. While not as extensively studied in the mouse, the Scn3aHyp model is notable for its incorporation of a hypermorphic mutation that causes increased channel expression and enhanced activation, leading to heightened neuronal excitability and increased seizure susceptibility (i.e., in response to proconvulsant stimuli) [47].

### Potassium channel models

#### **KCNQ2/3 (Kv7.2/7.3**)

KCNQ2 and KCNQ3 encode Kv7.2 and 7.3 channels (Figure 3), respectively, and generate M-current—a critical regulator of neuronal excitability. Pathogenic variants in KCNQ2/3 genes are associated with a spectrum of neurological disorders, primarily characterized by neonatal-onset epilepsy ranging from mild, self-limited familial neonatal epilepsy (SLFNE) to severe developmental and epileptic encephalopathy (DEE), often accompanied by intellectual disability [48]. Transgenic mouse models predominantly demonstrate the impact of LoF variants. The conditional KCNQ2 G279S model expresses a dominant-negative pore mutation (Figure 1B) restricted to the nervous system, with phenotypic severity depending on the timing of transgene expression [49]. Similarly, the Kcnq2 T274M variant in the selectivity filter region exhibits early generalized seizures and reduced lifespan followed by age-dependent amelioration of seizures, yet it demonstrates persistent deficits in spatial learning and memory [80]. The Kcnq2 A306T variant in the S6 segment of the pore domain (Figure 1B) exhibits channel gating defects where significant M-current reduction causes early-onset seizures and neurodevelopmental impairment [50]. The Y284C variant (ion pore region) generated using kick-in technology showed spontaneous seizures in homozygotes with reduced M-current [51]. In the S559A (C-terminus) model, disrupted phosphorylation alters channel function and seizure susceptibility, highlighting the importance of channel regulation [52]. Conditional knock-in mutations G279S and M547V (Figure 3B) have proved particularly valuable in demonstrating how M-current reduction in different neuronal populations affects seizure development [53,81]. Recent studies have also examined KCNQ2/3 GoF variants, revealing cell-type specific effects where the same GoF mutations can have differential impacts on excitability in CA1 versus cortical L2/3 pyramidal neurons, highlighting the complexity of GoF/LoF relationships in different neuronal populations [54].

#### **KCNA1 (Kv1.1**)

Mutations in the KCNA1 gene (encoding Kv1.1) cause a variety of human diseases, including epilepsy and episodic ataxia type 1 (EA1) [55]. Epilepsy or seizure-related variants tend to cluster in the **S1/S2 transmembrane domains** and in the **pore region** of Kv1.1, whereas EA1-associated variants occur along the whole length of the protein (Figure 1B). Complete knockout mice exhibit severe temporal lobe seizures and premature death [82], while conditional models with neuron-specific deletion of Kcna1 are associated with epilepsy, premature death, and cardiorespiratory dysregulation [57]. The V408A model replicates human episodic ataxia type 1 with associated seizures through dominant-negative suppression of channel function [58].

#### **KCNB1 (Kv2.1**)

Pathogenic variants in KCNB1 (encoding Kv2.1) are associated with an encephalopathy characterized by developmental delay and a variety of seizure types including myoclonic, tonic, and tonic-clonic seizures, often presenting as a DEE early in infancy [83]. These conditions can also manifest with neurodevelopmental disorders like autism spectrum disorder. While not exhibiting early mortality, Kcnb1 knockout mice demonstrate spontaneous seizures, neuronal hyperexcitability, increased startle responses, deficits in spatial learning, reduced anxiety-like behavior, and hyperactivity [84]. The G379R knock-in variant in pore-forming loop (P-loop) connecting the S5 and S6 transmembrane segments of the Kv2.1 channel mimics human epileptic encephalopathy by causing dominant-negative loss of channel function, which includes reduced potassium conductance and leads to neuronal hyperexcitability and spontaneous seizures [63]. Heterozygotes and homozygotes with the R306C variant in the S4 voltage-sensing transmembrane domain exhibit pronounced hyperactivity, higher seizures susceptibility, and frequent, long runs of spike wave discharges on EEG [83].

#### **KCNT1 (K_Na_1.1 or KCa4.1/SLACK**)

KCNT1, a sodium-activated potassium channel, regulates neuronal excitability through effects on resting membrane potential and post-spike hyperpolarization [65]. Pathogenic variants in KCNT1 can manifest as severe early-onset epileptic encephalopathies like malignant migrating focal seizures of infancy (EIMFS) and autosomal dominant nocturnal frontal lobe epilepsy (ADNFLE), often characterized by severe seizures with variable age of onset and cognitive impacts [66]. Kcnt1 knock-out mice exhibit a lower seizure susceptibility compared with WT mice, as well as deficits in spontaneous motor activity, learning abilities, and enhanced peripheral sensitivity to neuropathic pain [85]. The R455H model (analogous to the recurrent human variant R474H in the C-terminus) produces interictal discharges and electrographic seizures in heterozygous mice, with homozygous knock-in mice being stillborn [85]. The Y777H model (corresponding to the recurrent human variant Y796H) exhibits spontaneous tonic and generalized tonic-clonic seizures in homozygous mice, while heterozygous mice show seizures only rarely [62]. In contrast, both heterozygous and homozygous mice with the L437F GoF variant (channel pore region) show increased seizure susceptibility to proconvulsants but lack spontaneous seizures [86]. This variable penetrance reflects a common limitation in potassium channel epilepsy models, where homozygous expression is often required to observe seizure phenotypes that occur with heterozygous variants in humans [62]. Despite this caveat, the models demonstrate that K_Na_1.1 GoF variants result in seizure susceptibility primarily by dampening interneuron excitability rather than increasing pyramidal neuron excitability, producing generally milder phenotypes in heterozygous mice than seen in children with KCNT1-associated EIMFS [86].

#### Other Kv channel models

KCND2 encodes Kv4.2, a major pore-forming subunit involved in the somatodendritic subthreshold A-type potassium current channel. A reduction in the number of channels on the cell membrane of heterotopic neurons is associated with increased excitability and reduced seizure thresholds, particularly in cases associated with brain malformations [87]. Heterozygous Kcnd2 knockout mice revealed the importance of Kv4.2 in regulating neuronal network excitability and dendritic spine morphology [18]. These models demonstrate that even partial loss of Kv4.2 function significantly disrupts the normal balance between excitation and inhibition at the network level, with morphological changes in dendritic spines contributing to altered synaptic integration [88]. KCNV2 (Kv8.2) contributes to seizure susceptibility through distinct effects on neuronal firing patterns [89].

## Model-based insights into disease mechanisms

### Neuronal mechanisms

#### Cell-type specificity

Mouse models reveal distinct cell-type specific effects for different voltage-gated ion channels. SCN1A dysfunction primarily affects GABAergic interneurons expressing parvalbumin (PV) and somatostatin (SST) [90], although interneurons expressing vasoactive intestinal peptide (VIP) have also been shown to be dysfunctional in DS [91]. In contrast, SCN2A and SCN8A GoF models predominantly affect excitatory neurons, with Nav1.2 showing particular importance in pyramidal neurons during early development [92], and Na_V_1.6 playing a key role in hippocampal pathology in the N1768D model. Of note is the finding that Nav1.2 deficiency in the SCN2A^gtKO^ model results in hyperexcitability of principal medium spiny neurons (MSNs) in the striatum and pyramidal neurons in the medial prefrontal cortex (mPFC) [72].

Expression studies in developing brains of WT C57BL/6 mice indicate that Nav1.6 is primarily localized on neurons, with both astrocyte and neuronal protein levels steadily increasing from prenatal to postnatal stages, oligodendrocytes showing prominent expression postnatally, and microglia exhibiting low-intensity expression throughout development [45]. Single-cell RNA sequencing of hippocampal cells in the N1768D model found that Scn1a*,* Scn2a*,* Scn3a*,* and Scn8a were all expressed to varying levels in hippocampal excitatory and inhibitory neurons and oligodendrocyte precursor cells, with Nav1.2 and Nav1.6 also expressed in astrocytes [46]. The same study discovered that seizure onset was associated with many more gene expression changes in dentate gyrus granule cells than in any other hippocampal cell types. The SCN8A R1872W model revealed that expression of mutant protein in excitatory neurons alone suffices to cause seizures and lethality [35,76]. The paralogous SCN2A variant R1882Q predominantly affects pyramidal neurons causing early seizures [4], while K1422E shows broader effects across excitatory neuron populations [24]. In the case of the SCN8A R1872W model, parvalbumin interneuron dysfunction appears in addition to excitatory neuron effects, suggesting deficits in both excitatory and inhibitory synaptic transmission [93].

Potassium channels show complementary cell-type-specific patterns. KCNQ2 variants predominantly affect hippocampal and cortical neurons [59,61], where M-current regulation of AP generation and firing patterns is crucial. Pathogenic KCNA1 variants affect multiple neuron types, with the V408A model demonstrating how reduced potassium current across diverse neuronal populations leads to stress-induced seizures [55]. KCNB1 mutations (S379R, T374I) affect both excitatory and inhibitory neurons, producing complex circuit dysfunction in DEE [94,95]. In the KCNT1 (Slack) model, homozygous mice with the Y796H variant show reduced excitability of ‘non-fast spiking’ GABAergic neurons’ and enhanced, homotypic connectivity in both excitatory and inhibitory neurons [62]. Similarly, parvalbumin-positive hippocampal interneurons in heterozygous and homozygous mice with the L437F variant exhibit blunted evoked AP firing consistent with impaired inhibitory neurotransmission. These results support the paradoxical finding that GoF variants increase network excitability despite expression in inhibitory neurons [86].

#### Circuit-level effects

Studies across models reveal complex patterns of circuit disruption affecting both local and long-range connectivity. In SCN1A models, complex compensatory mechanisms maintain some network function during spontaneous cortical dynamics *in vivo* [96] (see below), although both SST- and PV-expressing cortical interneurons show impaired excitability leading to hyperexcitable networks [97]. Corticohippocampal circuits demonstrate significant dysfunction, with structural and functional deficits in the dentate gyrus network coinciding with seizure emergence [30,98]. SCN2A mutations produce network alterations that vary based on variant type and developmental timing, with studies of cortical circuits revealing that Nav1.2 dysfunction affects both local and long-range connectivity [92,99]. The Q54 model demonstrates how increased persistent sodium current in hippocampal neurons leads to circuit hyperexcitability [31], while strain differences in disorder severity correlate with hippocampal CaMKII activity (see below) [100]. Motor symptoms link to cerebellar circuits in the SCN8A T767I GoF model, demonstrating how distributed circuit effects contribute to complex phenotypes [37]. Similarly, Scn1b null mice demonstrate ataxia linked to cerebellar Purkinje cell hypoexcitability, with reduced sodium currents and altered firing patterns that may contribute to both motor dysfunction and seizure exacerbation [101]. Cell-type-specific studies have shown that Scn8a loss from cortical excitatory neurons increases seizure resistance, while loss from thalamic reticular nucleus inhibitory neurons can trigger absence seizures through altered thalamocortical synchrony [40].

Potassium channel dysfunction produces distinct but overlapping circuit effects. Conditional knockouts reveal varying severity based on the specific circuit elements affected [59]. KCNQ2 DEE models reveal early impacts on neural circuit formation that could have long-lasting effects [102]. KCNA1 complete knockout mice exhibit severe temporal lobe seizures and premature death, establishing the channel’s fundamental importance in circuit regulation [82]. The V408A knock-in model replicates human episodic ataxia type 1 with associated seizures through dominant-negative suppression of channel function, particularly affecting cerebellar circuits [58]. Recent studies of the conditional knockout of Kcna1 have found that the absence of Kv1.1 in forebrain corticolimbic circuits is sufficient to induce spontaneous seizures, premature mortality, and cardiorespiratory dysfunction [103]. These results also point to corticolimbic excitatory neurons as critical neural substrates in sudden unexplained death in epilepsy (SUDEP) and affirm seizure-related respiratory and cardiac failure as a likely cause of death.

#### Developmental aspects and temporal progression

The timing of channel expression and dysfunction critically influences both normal development and disease progression. For SCN1A, clear correlations exist between channel expression timing, epilepsy onset, and sudden death risk [104]. Developmental changes in the hippocampal CA1 circuit have been traced to both horizontal stratum-oriens (SO) interneurons and pyramidal neurons, with reduced function of SO interneurons persisting from the pre-epileptic through the stabilization stages and the greatest functional impairment observed during the severe stage of DS [32]. Interestingly, opposing changes were detected in CA1 excitatory neurons, indicating that the developmental trajectory of this disease is governed by reciprocal functional changes in both excitatory and inhibitory neurons [32]. Genetic background may also be an important modulating factor in the temporal progression of DS (see below) [105].

Nav1.2 plays distinct roles at different developmental stages, with especially important functions during early postnatal development. Nav1.2 haploinsufficiency in knockout mice causes an autistic-like phenotype that attenuates with age [99]. The A263V model demonstrates how early alterations in channel function and dendritic excitability can lead to both epilepsy and autism-like features [74,106]. For SCN8A, activation studies of the conditional R1872W model reveal a clear threshold effect, where spontaneous seizures emerge when mutant transcript reaches 8% of total Scn8a transcript [107]. All mice develop seizures at 50% mutant transcript expression, suggesting strong resistance of the normal adult brain to seizure development until a critical threshold is reached.

Potassium channel mutations show particularly severe developmental consequences. KCNQ2 mutations affect early neural circuit formation and function, with knock-in mutations producing spontaneous generalized seizures and cognitive impairment [80,102]. The conditional KCNQ2 Y284C model exemplifies how temporal control of channel dysfunction can reveal distinct developmental effects; this model expresses a dominant-negative pore mutation restricted to the nervous system, with phenotypic severity depending on the timing of transgene expression. Early expression leads to epilepsy, behavioral hyperactivity, and cognitive deficits, while later expression produces more limited effects [49]. Similar developmental impacts are seen in the KCNQ2 T274M knock-in model, which exhibits early generalized seizures and reduced lifespan, followed by age-dependent amelioration of seizures but persistent deficits in spatial learning and memory [80].

Pre-seizure alterations in neuronal excitability and circuit function emerge early in development across both channel types. For sodium channels, these include altered CA1 pyramidal cell properties [108] and behavioral abnormalities in the SCN1A A1783V model where motor impairment and hyperactivity manifest before seizure onset [109]. In potassium channel models, KCNQ2 dysfunction leads to progressive circuit changes before seizure onset, with both electrophysiological and behavioral alterations preceding clinical manifestations [80]. The progression from early dysfunction to spontaneous seizures and cognitive impairment demonstrates how channel dysfunction may be a trigger for cellular pathologies in the lead up to seizure onset. Notably, attenuating M-current suppression can prevent both acute seizures and subsequent circuit reorganization, highlighting the relationship between early dysfunction and disease progression [81]. This temporal progression across different channel types suggests common developmental mechanisms and vulnerability periods where early intervention might be most effective.

### Non-neuronal mechanisms

#### Oxidative stress and mitochondrial dysfunction

Both sodium and potassium channelopathies have been shown to be associated with significant metabolic alterations. Mice heterozygous for the Kcnt1 R455H GoF variant have increased expression of proteins in electron transport chain (ETC) complexes III, IV, and V of the inner mitochondrial membrane, indicating a dynamic relationship between Slack channels and the bioenergetics of neuronal mitochondria [110]. In sodium channel models, male Scn1a^+/-^ mice show cardiac mitochondrial alterations with significant changes in mitochondrial respiration and reactive oxygen species (ROS) production, which may have significance for risk of SUDEP [111]. Scn2a Q54 mice may exhibit a heightened response to oxidative stress as evidenced by increased levels of hippocampal CaMKII activity, which leads to enhanced persistent current and depolarized channel inactivation [100]. These results are consistent with studies of SCN2A patient-induced pluripotent stem cell (iPSC)-derived neurons demonstrating dysregulation of oxidative phosphorylation pathways and activation of calcium signaling and neurotransmission [112], as well as those showing a relationship between SCN2A expression and oxidative stress in the cortexes of patients with temporal lobe epilepsy [113]. Hippocampal gene expression profiling in the pre-seizure SCN8A N1768D model reveals evidence for early oxidative stress, with males showing further cellular stress in response to increasing neuronal hyperexcitability [114].

#### Glial cell activation

Both sodium and potassium channelopathies exert significant effects on glial function, which plays an important role in epileptogenesis [115]. Spontaneous Ca^2+^ spiking was found to be significantly faster in the astrocytes of Scn1a^+/-^ mice, indicating that Ca^2+^ dynamics may be altered in both astrocytes and neurons in the pathogenesis of DS [116]. Impaired microglial function has been observed in Scn1a E1099X mice, likely resulting in an excess of immature synaptic connections, neuronal hyperexcitation, and the formation of abnormal neural circuits in the hippocampus of this model of DS [117]. SCN2A dysfunction, while primarily affecting neurons, produces secondary effects in oligodendrocyte progenitor cells, which express both Nav and Kv channels that potentially contribute to AP generation [118]. SCN8A models demonstrate significant astrocyte reactivity [119,120], with strong astrogliosis after seizure onset [120]. Hippocampal reactive astrocytosis is associated with epileptic seizures, cognitive and behavioral deficits, and neuronal loss resulting from restricted axonal surface expression of Kv7 channels in the KCNQ2 M547V model [53].

#### Blood–brain barrier dysfunction and neuroinflammation

Building on established links between blood**–**brain barrier (BBB) dysfunction and epilepsy [121], studies of both sodium and potassium channelopathies have revealed specific mechanisms of BBB disruption. Proteomic analysis of mice carrying the Scn1a A1783V variant revealed distinct protein signatures affecting synaptic function, cellular stress responses, inflammatory processes, and BBB dysfunction prior to the onset of spontaneous seizures in this model of DS [122]. Single-nucleus RNA profiling further revealed seizure-induced transcriptional changes in multiple molecular pathways, suggesting that seizures themselves trigger additional pathogenic mechanisms [123]. Direct measurements of barrier permeability in the SCN8A N1768D model revealed that BBB dysfunction precedes seizure onset [114] and is accompanied by early transcriptional changes affecting neurotransmitter release, synaptic vesicle trafficking, and ion transport [124,125].

#### Sleep and systemic effects

The reciprocal relationship between sleep and epilepsy has been reported by numerous clinical studies—confirmation in mouse models could lay the ground for mechanistic insights. Electroencephalographic studies uncovered abnormal sleep in DS mice, including reduced sleep spindles, increased brief wakes, reduced delta wave power in rapid-eye-movement (REM) sleep, and numerous interictal spikes in non-rapid-eye-movement (NREM) sleep [126]. Indeed, results show that sleep disorder in Scn1a^+/-^ mice arises from loss of NaV1.1 channels in forebrain GABAergic interneurons. EEG patterns in the SCN1A GEFS+ R1648H model revealed sleep deficit as evidenced by increased wakefulness and reduced NREM and REM sleep during the dark phase, pointing to the role of SCN1A in sleep regulation and seizure generation [127]. Scn2a deficiency has also been shown to result in increased wakefulness and reduced NREM sleep, disrupting the firing pattern of spontaneously firing neurons in the suprachiasmatic nucleus (SCN) containing region, which is involved in circadian rhythms [128]. This was reflected at the molecular level as genes in the circadian entrainment pathway were found to be dysregulated, which may provide novel targets for therapeutic interventions. Non-invasive piezoelectric monitoring in the N1768D SCN8A model revealed disrupted sleep architecture coinciding with seizure onset, with exacerbation during seizure bouts and recovery during longer inter-bout periods [129]. In the KCNT1 Y777H model, nocturnal tonic-clonic seizures were found to induce more NREM sleep and suppress REM sleep, resulting in altered sleep architecture [130]. Importantly, seizure number was anti-correlated with the amount of REM sleep. Beyond sleep, systemic effects extend to peripheral systems, with Nav1.6 playing crucial roles in proprioceptive signaling [131]. The loss of Nav1.6-mediated proprioceptive feedback causes non-cell-autonomous impairments in end-organs and skeletal muscle, while potassium channel dysfunction can affect cardiac function, autonomic regulation, and SUDEP [103].

## Integration of mechanistic insights: toward a synthetic model of ion channel epileptogenesis

**Figure 4:**
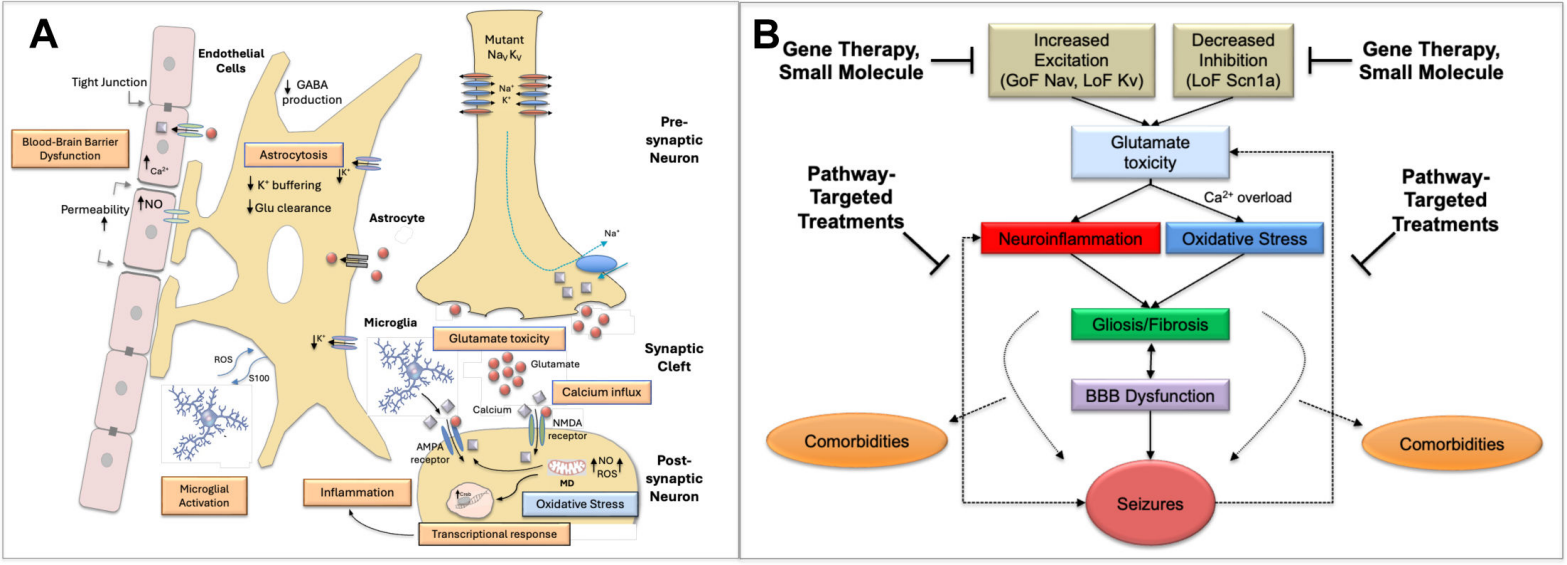
Unifying conceptual model of epileptogenesis associated with ion channelopathies. **A**. Downstream ‘injury’ cascade (see text). **B**. Synthetic model of epileptogenesis triggered by dysfunctional nav and kv channels and targeted and off target therapeutic approaches. Neuronal hyperexcitability triggers a sequential chain including glutamate excitotoxicity, calcium overload, oxidative stress/mitochondrial dysfunction, inflammatory responses, glial activation, BBB dysfunction, fibrosis and seizures [132]. The model includes several feedback loops and highlights the importance of neuroinflammatory processes [133]. *Points with feed forward loops on which therapeutic compounds may intervene. GABA, gamma-aminobutyric acid; Glu, glutamate; Kv, potassium channel; MD, mitochondrial dysfunction; Nav, sodium channel; NO, nitric oxide; ROS, reactive oxygen species.

Integration of findings across Nav and Kv channel models reveals many common pathological processes in the chain of events leading from initial channel dysfunction to the onset of chronic seizures. In this section, these processes are brought together and integrated into a conceptual model of ion channel-based epileptogenesis (Figure 4). A key feature of the model is the multiple feedback loops leading to self-sustaining pathological cycles. The initiating events involve alterations in channel function due to pathogenic alterations in sodium (SCN1A, SCN2A, SCN8A) or potassium (KCNQ2, KCNQ3, KCNT1, KCNA1) channel genes, which increase neuronal excitability through distinct mechanisms [1,2].

These initial impairments in channel function are the precursors to a downstream ‘injury cascade’ involving many of the processes in Figure 4A. Increased neuronal firing, whether directly through hyperexcitation or indirectly through disinhibition, leads to enhanced glutamate release from presynaptic terminals [7,134]. The excessive glutamate release triggers pathological activation of postsynaptic AMPA and NMDA receptors, resulting in excessive calcium influx [125]. This calcium overload represents a critical point in the pathological cascade, initiating multiple destructive processes in postsynaptic neurons [135]. A key consequence is increased production of ROS, particularly through disruption of mitochondrial ETC function [136]. The combination of increased nitric oxide production through activated nNOS and elevated superoxide leads to peroxynitrite formation, causing widespread oxidative damage to cellular components [137]. Table 2 provides an overview of models that have provided evidence for the existence of ‘downstream injury’ processes and sleep disruption.

**Table 2:** Downstream injury processes in ion channel epileptogenesis mouse models

Process or consequence	SCN1A	SCN2A	SCN8A	KCNQ2/3	KCNA1	KCNB1	KCNT1
Enhanced glutamate release	Scn1a^+/-^ [116]	Q54 [31] R1882Q [4]	N1768D [114] R1872W [35,76]	Y284C [49] T274M [47]	Knockout [82] V408A [57]	S379R [63]	na
AMPA/NMDA receptor activation	na	na	N1768D [114,125]	na	na	na	R455H [110]
Excessive calcium influx	A1783V [122]	Q54 [100]	N1768D [114,125]	Y284C [49]	na	na	na
ROS production	Scn1a^+/-^ [111]	Q54 [100]	N1768D [114]	na	na	na	na
Nitric oxide/peroxynitrite	na	na	N1768D [114,125]	na	na	S379R [63]	na
Mitochondrial dysfunction	Scn1a^+/-^ [111]	na	N1768D [114,125]	na	na	na	R455H [110]
Glial cell activation	Scn1a^+/-^ [116] E1099X [117]	na	N1768D [119,120]	M547V [53]	na	na	na
Neuroinflammation	A1783V [122]	na	N1768D [114,125], R1872W [35]	na	na	na	na
BBB dysfunction	A1783V [122]	na	N1768D [114]	na	na	na	na
Circuit reorganization	Scn1a^+/-^ [96]	Q54 [31]	R1627H [40]	na	Knockout [82] V408A [57]	na	na
Sleep disruption	Scn1a^+/-^ [126] R1648H [127]	Scn2a^-^deficient [128]	N1768D [129]	na	na	na	Y777H [130]

Mitochondrial dysfunction emerges as a central hub in the pathological cascade (Figure 4A). Calcium overload triggers the mitochondrial permeability transition, leading to loss of membrane potential, compromised ATP production, and release of pro-apoptotic factors [110,125,136,138]. The resulting bioenergetic crisis creates a destructive cycle where impaired energy metabolism further compromises cellular calcium homeostasis [125]. Sustained cellular stress activates both astrocytes and microglia, initiating neuroinflammatory responses that amplify the pathological cascade [125,139,140]. Indeed, significant alterations in astrocyte calcium signaling and microglial responses have been documented in several models [116,117,120]. Reactive astrocytes undergo morphological and functional changes affecting glutamate uptake and potassium buffering [140,141] (Figure 4A). This is particularly evident in SCN8A models, where astrogliosis correlates with reduced Kir4.1-mediated currents, suggesting that impaired ion and neurotransmitter handling may exacerbate hyperexcitability [120]. Activated microglia release pro-inflammatory cytokines including IL-1β, TNF-α, and IL-6, contributing to a pro-inflammatory environment [142,143].

The recognition of the importance of astrocytic-neuronal communication in regulating excitability, as well as cross-talk between astrocytes and brain endothelial cells, focuses attention on the BBB as another central hub in the model (Figure 4A). Indeed, increased permeability of the BBB can begin before seizure onset in some models [114], providing evidence for a primary role of BBB dysfunction in epileptogenesis [121,124,144]. Inflammatory mediators affect tight junction proteins and increase barrier permeability, allowing infiltration of peripheral immune cells and development of vasogenic dysfunction [125,145]. This creates an additional destructive cycle where increased inflammatory mediator penetration triggers further barrier disruption, promoting progressive deterioration of neurovascular function.

The convergence of these pathological processes—oxidative stress, mitochondrial dysfunction, neuroinflammation, and BBB permeability—creates multiple reinforcing cycles that maintain cellular damage independently of the initial channel dysfunction [114,125]. Figure 4B summarizes these pathological processes triggered by dysfunctional Nav and Kv channels in the form of a synthetic model of epileptogenesis. The activation of these interactive and self-sustaining processes, particularly as they become entrenched over time, raises the question of whether channel-directed therapies alone will be sufficient to mitigate and reverse the disease process.

## Therapeutic implications

The identification of common pathological disease mechanisms among mouse models involving both channel dysfunction and downstream pathological processes may reveal novel therapeutic targets. In this section, we review current *gene targeted* and *pathway-targeted* therapeutic strategies (Figure 4B) before addressing the question of whether optimal strategies may require the targeting of multiple points in the injury cascade [146,147].

### Targeted therapies

#### Antisense oligonucleotide (ASO) approaches

ASO therapy has emerged as a versatile approach across multiple channel types and mutation mechanisms [reviews: [148,149]]. The targeted augmentation of nuclear gene output (TANGO) approach, which uses ASOs to enhance expression of the functional allele, reduced intrinsic excitability of parvalbumin-positive (PV) inhibitory interneurons [150], and reduced both seizure frequency and SUDEP incidence in the Scn1a^+/-^ models of DS [151] (Table 3). While treatment of juvenile mice (i.e., close to the time of seizure onset ~ P14-21) was clearly not as robust as was observed when animals were treated as neonates (i.e., at P2), a single intracerebroventricular (ICV) injection of a related ASO at P2 resulted in long-lasting increases in brain Nav1.1 protein and prolonged survival in DS mice [152]. This suggests some degree of reversibility in the DS phenotype even after seizure establishment. ASO treatment targeting SCN2A has also demonstrated success by decreasing overall channel expression in mice carrying the R1882Q transgene [4]. When given at P1, the Scn2a ASO treatment prolonged lifespan and rescued the seizure phenotype; however, this effect was lost after 60 days when the ASO was cleared from the brain and the seizure phenotype reemerged. This suggests that repeated administration of the ASO in later development is needed to maintain a therapeutic level of Scn2a ASO (Table 3).

**Table 3:** ASO and viral treatment studies in mouse models

Gene	Model	Mouse model context	Treatment timing	Effects on seizures and lifespan (Early)	Effects on seizures and lifespan (Late)	Citations
**ASO gene therapy**						
SCN1A	Scn1a^+/-^	Dravet syndrome, loss-of-function	P2, P14	Increased Nav1.1; improved survival, reduced seizures	Less robust effects, prolonged survival in fewer mice	[151]
	Scn1a^+/-^		P2	Reduced excitability PV inhibitory interneurons		[150]
	Scn1a^+/-^		P2	Restored PV function, reduced seizure frequency, and SUDEP		[152]
SCN2A	R1882Q	Gain-of-function, autism, and epilepsy	P1, P14-16	Normalized hyperexcitability, reduced seizures, extended lifespan; interictal spikes return over time	Extended survival, reduced seizure frequency	[4]
SCN8A	N1768D	Gain-of-function, severe epilepsy	Within 1–3 days after seizure onset, repeated at 1 m intervals		Reduced seizure frequency, less impact on lifespan	[153]
	R1872W	Gain-of-function, severe epilepsy	P2, P30	Seizure reduction, prolonged survival	Increased survival	[154]
KCNT1	P942L	GoF, EIMFS	P2, P40	Reduced seizure, prolonged survival, improved behavioral performance	Reduced seizure, prolonged survival, improved behavioral performance	
			P2, repeat at P30		Further extension in survival, improved behavior	[155]
**Viral gene therapy**						
SCN1A	Scn1a^+/-^	LoF, Dravet syndrome	P1	Increased Nav1.1 expression, seizure reduction, prolonged lifespan ( > 1 yr)		[156]
SCN2A	Scn2a^gtKO^	LoF, behavioral deficits	P2	Restoration of Scn2a expression, reduced hyperexcitability, normalized behavioral impairments		[157]
SCN8A	R1872W	GoF, severe epilepsy	P1	Long term protection against seizures and death (12 m)		[153]

Given that deleting the Scn8a transcript is lethal, dosing of an ASO must be carefully adjusted to partially reduce Scn8a mRNA while maintaining sufficient levels to observe a therapeutic effect (i.e., the Goldilocks effect) [158]. Treatment of the conditional SCN8A R1872W mouse model with an ICV injection of an ASO at P2 (i.e., prior to the onset of symptoms) led to a reduction in Scn8a transcript by 25 to 50%, delayed seizure onset, and prolonged survival up to seven  weeks of age [154]. Mice receiving a second dose of ASO at P30 benefitted by a further increase in median survival of up to approximately nine weeks, indicating that repeated ASO administration might be effective as a long-term strategy. In the case of ASO treatment of SCN8A N1768D mice, repeated administration (i.e., every 1.0–1.5 months) initiated shortly after seizure onset was required to maintain long-term survival and reduced seizure frequency (i.e., during a 12 month observation period) [153] (Table 3). In contrast, administration of a single dose of AAV10-short hairpin RNA (shRNA) at P1 provided long-term protection against seizures and death in the SCN8A R1872W model [153]. These results suggest that early intervention in the neonatal period may provide more durable therapeutic benefit than treatment initiated after seizure onset. This points to the possibility that once the seizure cascade is established, continuous suppression of the mutant Nav1.6 channel may be necessary for sustained benefit. Interestingly, reduction in the expression of SCN8A has been shown to extend survival in Scn1a^+/-^ haploinsufficient mouse models of DS and channelopathies involving Kcna1 and Kcnq2 [153,154,159], highlighting the broader therapeutic potential of addressing hyperexcitability through modulation of Nav1.6 expression.

ASO efficacy was evaluated in homozygous Kcnt1 P924L mice, a DEE model exhibiting spontaneous seizures, behavioral abnormalities, and early death. Following a single ICV injection of the Kcnt1 ASO at P40, mice showed a nearly 90% reduction in Kcnt1 mRNA levels, resulting in near-complete abolition of seizures, prolonged survival, and improved performance on behavioral tests [155] (Table 3). ASO administration at neonatal age was also effective in controlling seizures and extending the life span. Interestingly, treatment of heterozygous Slack-R455H mice with an ASO to reduce expression of Slack channel was associated with a reduction in the overexpression of mitochondrial ETC complex III, IV, and V proteins [110]. A study using ASOs to reduce Kcnt1 expression prolonged survival of both Scn1a and Scn8a mutant mice, supporting KCNT1 as a therapeutic target for treatment of other GoF channelopathies [160].

#### Viral gene therapy approaches

Cell-selective AAV-mediated permits precise therapeutic intervention in affected neural circuits, an approach that has rescued mortality and seizure phenotypes in SCN1A DS models. A single ICV injection (in P1 mice) of an AAV serotype 9 (AAV9) vector engineered to up-regulate SCN1A expression levels within GABAergic inhibitory interneurons led to increased SCN1A mRNA transcripts, specifically within GABAergic inhibitory interneurons, and Nav1.1 protein levels in the brain [156]. This was associated with a significant decrease in the occurrence of spontaneous and hyperthermia-induced seizures and prolonged survival for over a year without adverse effects. Injection of an AAV vector coding for the multifunctional beta1 sodium channel auxiliary subunit (AAV-NaVbeta1) in neonatal mice mitigated the higher mortality displayed by untreated Scn1a^+/-^ females, as well as the elevated motor hyperactivity, spontaneous seizure rate, and behavior deficits exhibited by untreated males [161].

Unlike the case of N1768D mice requiring repeated ASO administration to effectuate long-term protection, Scn8a R1872W neonatal mice treated a single time with an AAV10-shRNA were protected over a period of 12 months [153]. A similar lesson was learned in the application of AAV-mediated gene replacement in SCN1B-DS mice: reduced seizures and prolonged lifespan were observed in treating neonatal but not juvenile mice, highlighting the critical importance of intervention at early time points to obtain therapeutic efficacy [162]. Regarding the treatment of LoF phenotypes, an AAV strategy was used in the Nav1.2-deficient Scn2a^gtKO^ model that resulted in normalization of aberrant basal activity and social deficits associated with intrinsic hyperexcitability in striatal MSNs [157]. Interestingly, these experiments restored physiological Scn2a activity and reversal of social deficits both by use of viral strategies and by pharmacological enhancement of GABA_A_ receptor function [157]. Recently, advances in viral gene therapy have addressed the packaging limitations for large genes like SCN1A. A dual-vector approach using split-intein technology enabled interneuron-specific SCN1A gene replacement, providing strong protection against mortality and seizures in DS mouse models (i.e., Scn1afl/+;Meox2-Cre and Scn1a+/R613X) while avoiding the adverse effects observed with pan-neuronal expression [163].

#### CRISPR and base editing approaches

Beyond traditional ASO approaches, precision genome editing strategies have emerged as promising alternatives. For GoF variants, allele-specific CRISPR editing of the SCN8A N1768D allele achieved 25-33% editing efficiency throughout the brain, sufficient to rescue lethality and seizures [164]. Base editing offers particular precision for these pathogenic mutations: adenine base editor therapy targeting SCN8A R1872W achieved ~ 30% conversion efficiency and significantly improved survival, seizures, and behavioral phenotypes [165].

For haploinsufficiency conditions, CRISPR activation (CRISPRa) strategies can up-regulate the remaining functional allele. In SCN1A haploinsufficiency, CRISPR/dCas9-mediated gene activation increased Nav1.1 expression in parvalbumin-positive neurons, ameliorating seizures and behavioral impairments even when applied after juvenile stages [166]. Similarly, CRISPRa treatment of Scn2a haploinsufficiency up-regulated the WT gene copy, restored electrophysiological deficits, corrected intrinsic and synaptic deficits in neocortical pyramidal cells, and protected adolescent mice against chemoconvulsant-induced seizures [167].

#### Small molecule channel modulators

PRAX compounds are functionally selective small molecules that inhibit persistent sodium current (I_NaP_) with preference over peak (I_Na_) compared with standard SCBs [147,168]. Chronic treatment of Scn8a N1768D/+ mice with GS967 resulted in lower seizure burden and complete protection from seizure-associated lethality without overt adverse effects [168], as well as prolonged survival in mice with global expression of R1872W [35]. Prax330 reduced both I_NaP_ and resurgent current (I_NaR_) and reduced synaptically-evoked APs in Scn8a N1768D+ (but not WT) subiculum neurons [169]. PRAX-562 exhibited anticonvulsant activity in multiple Nav (Scn2aQ54 and Scn8aN1768D) and Kv (K556E and Kcnc1 R320H) models indicating broad efficacy regardless of the underlying genetic basis [146]. Preferential targeting of I_NaP_ may represent a therapeutic advantage given the non-selectivity and generally poor tolerability of standard SCBs [147]. Similarly, retigabine has shown efficacy in KCNQ2 LoF models by enhancing potassium channel opening and restoring hyperpolarizing current [81].

Another form of selectivity has been achieved through the development of a first-in-class compound that specifically binds to Nav1.6 with much greater selectivity over other sodium channel isoforms. NBI-921352 is a state-dependent inhibitor—preferentially inhibiting activated channels (inactivated and open states)—that stabilizes inactivation and inhibits Nav1.6 currents (I_NaP_ and I_NaR_) [170]. This isoform selectivity provides inhibition of action-potential firing in glutamatergic excitatory pyramidal neurons, while sparing fast-spiking inhibitory interneurons, thus improving tolerability by avoiding the adverse effects that result from broad scale inhibition of other Nav isoforms expressed in multiple cell types and organs [171]. Oral administration of NBI-921352 prevented electrically induced seizures in the SCN8A N1768D model and prevented seizures at lower brain and plasma concentrations than commonly prescribed antiseizure medications (ASMs) [172].

## Pathway-targeted treatments

The potential of several alternative therapeutic approaches has emerged from the testing of natural products and repurposed compounds. Huperzine A, a naturally occurring acetylcholinesterase inhibitor that also serves as an antagonist of N-methyl-d-aspartate glutamate (NMDA) receptors, provides sustained protection against induced seizures in Scn1a^+/-^ mutant mice [173]. Treatment using nanoparticle encapsulation of oxytocin conferred sustained protection against induced seizures and restored normal social behavior in the same model of DS [174]. This effect may result from modulating the balance of neurotransmitters (i.e., by enhancing GABAergic activity while reducing glutamatergic activity). Cannabidiol (CBD) has also been demonstrated to attenuate seizures and social deficits in the Scn1a^+/-^ mouse model of DS [175,176], as well as in the mixed GoF/LoF SCN8A R1620L model [177]. The beneficial effects of CBD in the SCN1A model were shown to be mediated through antagonism of GPR55, a lipid-activated G protein-coupled receptor that modulates pain perception and inflammation within the nervous system [176]. A sex-specific response to CBD has been noted in the SCN8A N1768D model, suggesting some degree of hormonal involvement [129]. The finding of increased seizure resistance in Scn8a R1620L mice when treated with the non-selective beta-blocker carvedilol [178], and the discovery of the role of KCNT1 in regulating inner mitochondrial membrane proteins, highlight the potential of therapeutic targets independent of direct channel modulation and that may mitigate the effects of oxidative stress and mitochondrial dysfunction [110].

**Figure 5:**
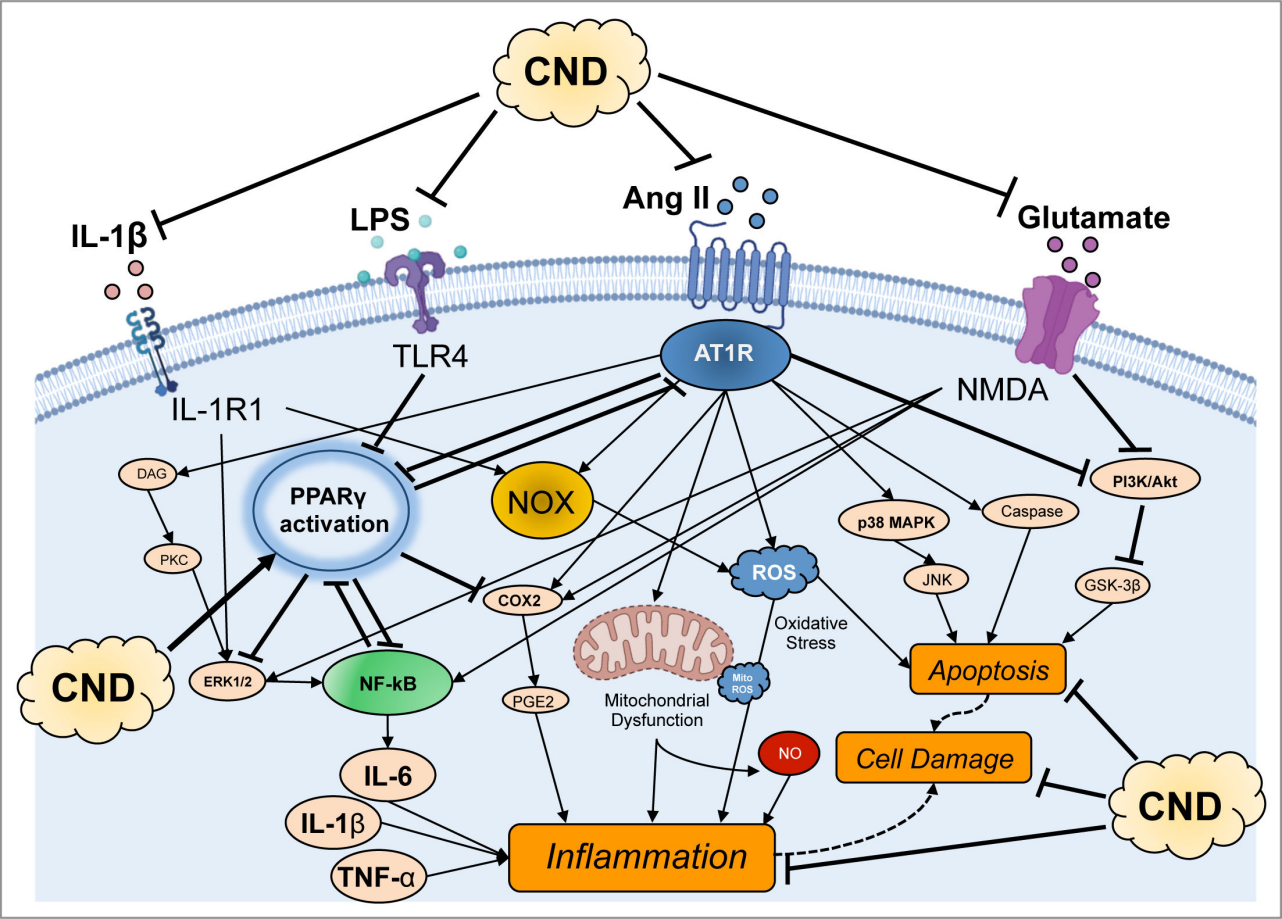
Overstimulation of Angiotensin II Type 1 Receptors (AT1Rs) increases inflammatory and apoptotic signaling. Mechanisms include interaction with membrane receptors and signaling pathways for injury factors (interleukin-1beta (IL-1β), lipopolysaccharide (LPS), Angiotensin II (Ang II), glutamate) leading to enhanced cell injury. Augmented Nicotinamide adenine dinucleotide phosphate oxidase (NOX) activation (e.g., via stimulation of the p47phox subunit of NOX2 by glutamate) and increased oxidative stress with enhanced formation of reactive oxygen species (ROS) leads to mitochondrial dysfunction with excessive formation of mitochondrial ROS, enhancing both inflammation and apoptosis. Additional inflammatory mechanisms include activation of cyclooxygenase-2 (COX2) with production of inflammatory prostaglandins (not shown), increased toxic levels of nitric oxide (NO). The result is increased intranuclear trafficking of transcription factors such as nuclear factor-kappaB (NF-kB) with enhanced production of pro-inflammatory, neurotoxic cytokines, such as IL-1β, tumor necrosis factor alpha (TNFa), and interleukin-6. Pro-apoptotic mechanisms include reduction in survival pathways such as the phosphoinositide-3-kinase/ protein kinase B/Akt (PI3K/Akt) and stimulation of the p38 mitogen-activated protein kinase (p38 MAPK) and c-Jun N-terminal kinase (JNK) (not shown). Further, AT1R overstimulation inhibits the neuroprotective peroxisome proliferator-activated receptor gamma (PPARγ). By blocking AT1R overstimulation, candesartan (CND) reduces expression of membrane receptors activated by injury factors (IL-1R1, TLR4, NMDA), their corresponding apoptotic and inflammatory signaling, and PPARγ inhibition. AT1R, angeniotensin II type 1 receptor; IL-1R1, interleukin-1 receptor type 1; NMDA, N-methyl-d-aspartate. Modified from [182].

Anti-inflammatory strategies have proven particularly effective in the SCN8A N1768D model. Post-seizure onset administration of candesartan—an FDA-approved angiotensin receptor blocker (ARB) indicated for hypertension—restores BBB function, reduces seizure frequency, and extends lifespan in juvenile and adult mice [132]. The efficacy of candesartan is likely due to its dual mechanism of action (MOA) as both an angiotensin type 1 receptor (AT1R) antagonist and a partial PPARγ agonist. ARBs are known to have powerful anti-inflammatory, anti-fibrotic, and antioxidant activities, which may involve both of these mechanisms [179–181] (Figure 5). In animal models of traumatic brain injury (TBI), AT1R blockade has been shown to reduce oxidative stress, stabilize the BBB, and prevent the development of seizures, in part by inhibiting signaling of the pro-fibrotic cytokine TGFβ [181]. Activation of widely expressed PPAR receptors leads to additional anti-inflammatory, neuroprotective, and metabolic effects by playing a role in reducing the production of ROS and promoting mitochondrial function [180].

## Combinatorial targeted approaches

Increasing evidence from mouse models suggests that purely channel-directed therapies, while crucial, may have limited efficacy once disease processes are established. This limitation stems from the development of self-perpetuating pathological cycles that exhibit independence from initial channel dysfunction, as demonstrated across multiple models [125] (Figure 4B). An important unanswered question is whether these processes are inherent to an underlying pathophysiology triggered by altered expression of Nav and Kv channels early in development. The establishment of complex cellular and network-level changes may require a multipronged approach to therapeutic intervention, combining targeted and pathway-based approaches. Early intervention with channel-directed therapies may be most effective in preventing the establishment of pathological cycles, as demonstrated in both sodium and potassium channel models [80,95,102,152,162,183]. However, once disease processes are established, simultaneous targeting of downstream pathways may become necessary (Figure 4B). This understanding has led to increased interest in combinations of single-target drugs and/or rationally designed multi-target drugs in the treatment and prevention of epilepsy [117,132,184,185].

## Ion channel compensation, genetic background effects, and novel therapeutic targets

### Compensatory mechanisms as therapeutic opportunities

Transgenic mouse models have revealed that ion channel dysfunction triggers compensatory responses that represent potential therapeutic targets [186]. Given the co-expression of Nav and Kv channels at the AIS (Figure 3) [187], loss of one channel type often leads to adaptive changes in others. Intercrossing Scn1a^+/-^ mice with Scn8a^med-jo/+^ (LoF) mice reduces seizures and increases survival compared with Scn1a^+/-^ mice alone [22], while antisense oligonucleotides targeting Scn8a reduce seizures in mouse models with Kv channel LoF mutations [159].

Nav and Kv channel interactions can influence AP generation in counterintuitive ways. Conditional deletion of Scn2a in pyramidal cells paradoxically increases excitability despite loss of sodium conductance [10]. This occurs because spike initiation shifts from the proximal AIS, where Nav1.2 normally co-activates Kv channels (Figure 3), to the distal AIS where Nav1.6 dominates without equivalent Kv channel engagement [9]. The result is a paradoxical increase in excitability, mainly due to impaired repolarization and enhanced firing [10]. Similarly, down-regulation of Kv channels can reverse pyramidal cell hypoexcitability in Nav1.2-deficient models [72], suggesting that targeting compensatory channels may restore functional balance. Developmental mechanisms may also occur such that sodium current density that is selectively reduced in GABAergic neurons later becomes elevated in pyramidal neurons [186]. The presence of these cellular and synaptic changes points toward homeostatic adaptations and complex network remodeling [188].

### Strain-specific background effects and modifier gene discovery

Epilepsy phenotypes vary dramatically across mouse strains, providing insights into genetic modifiers relevant to human disease variability. Scn1a^+/-^ mice show minimal phenotypes on 129 backgrounds but severe epilepsy with premature death on C57BL/6J backgrounds [41,105]. Even within C57BL/6 substrains, C57BL/6 N mice show milder seizures than C57BL/6 J mice [189], as the latter has a private mutation in the splice site of the Gabra2 gene that reduces expression of the WT protein to 25% of WT levels [190]. The cellular basis involves differential impairment of GABAergic interneuron excitability between strains [105].

Similar strain dependencies occur across multiple models: Scn2a Q54 variants show mild phenotypes on C57BL/6J but severe epilepsy on SJL/J backgrounds due to dominant modifier alleles [100,191], while Scn8a V929F variants produce more severe spike-wave discharges on C3HeB/FeJ compared with C57BL/6J backgrounds [192]. Strain dependency of features associated with LoF SCN8A models like absence seizures is also known to occur [193]. Kv channel models show similar strain-dependent effects on seizure susceptibility and survival [50,82].

Quantitative trait locus mapping and transcriptomic approaches have identified specific modifier genes [41]. In Scn2a Q54 mice, genome-wide mapping identified Kcnv2 as a potential modifier, suggesting Nav-Kv channel gene interactions modulate phenotype severity [191]. The gene encoding the alpha2 subunit of the aminobutyric acid type A receptor (Gabra2) modifies SCN8A phenotypes across strain backgrounds [194], while strain-dependent CaMKII activity differences affect Nav channel properties in Scn2a models [100]. The endocannabinoid system also contributes through strain-dependent cannabinoid receptor expression differences [175].

### Dynamic channel regulation and therapeutic targets

Beyond genetic compensation, post-translational modifications provide dynamic regulation that may offer therapeutic opportunities. Phosphorylation creates activity-dependent control of channel function, with different Nav channel isoforms showing distinct regulatory patterns. PKA phosphorylation of Nav1.2 at Ser573 reduces current amplitude and stabilizes slow inactivation [69,195], while Nav1.6 is dually regulated: enhanced by CaMKII phosphorylation [196] but reduced by p38 MAP kinase (Figure 1), the latter of which creates Nedd4 ubiquitin ligase binding sites that promote channel endocytosis during cellular injury [69,197].

Kv channel phosphorylation creates homeostatic mechanisms linking cellular activity and energy status to excitability. CDK5 phosphorylation of Kv2.1 produces hyperpolarizing shifts in voltage-dependent activation, while AMPK phosphorylation links cellular energy status to neuronal excitability through voltage-dependent shifts in both activation and inactivation [68,198] (Figure 2). These regulatory pathways represent potential targets for modulating channel function without directly targeting the primary channel defect. Consult Supplementary Table S1 for a detailed list of key phosphorylation sites and their physiological consequences.

Auxiliary subunits provide additional therapeutic opportunities by modifying channel gating, trafficking, and pharmacological sensitivity. Nav channel β-subunits alter gating kinetics and surface expression [67,199], while Kv channel auxiliary subunits dramatically expand functional diversity. KChIP and DPPX subunits modify Kv4 channel properties [17], while KCNE subunits alter Kv7 channel pharmacological sensitivity [200], suggesting that targeting auxiliary subunit interactions could provide isoform-specific therapeutic effects.

### Implications for precision medicine

These findings highlight the importance of genetic background in translating mouse model discoveries to human therapeutics. Strain-specific modifier genes may reveal new therapeutic targets while helping explain the variable expressivity characteristic of human genetic epilepsies [2]. The complex interplay between primary channel dysfunction, compensatory mechanisms, genetic modifiers, and dynamic regulation suggests that effective therapies may need to target multiple components of these networks rather than focusing solely on the primary channel defect. Understanding these relationships through transgenic mouse models provides a foundation for developing precision medicine approaches that account for individual genetic backgrounds and compensatory mechanisms in treating genetic epilepsies.

## Conclusion

While targeted genetic therapies for these channelopathies show great potential, they are still in early stages of development and are likely several years away from being readily available to patients due to the complexities of clinical trials, safety concerns, and the need for further research to optimize their effectiveness. In the meantime, continued studies of transgenic mouse models will advance our understanding of disease mechanisms, many of which may lead to the discovery of novel therapeutic targets. The integration of mechanistic insights across channelopathy models brings to light a few common principles. The initial channel dysfunction initiates multiple pathogenic cascades that can become self-perpetuating and independent of the original insult. For example, neuroinflammatory mechanisms can create positive feedback loops where inflammation promotes seizures, and seizures trigger further inflammation [201] (Figure 4). It has been posited that a pathogenic ‘tetrad’ of glutamate excitotoxicity, oxidative stress, inflammation, and BBB dysfunction characterizes the neurobiology of a variety of brain disorders, including epilepsy [132,202]. Indeed, the notion that seizures beget seizures can be traced back to the early 20th century [203].

Early treatment targeting channel dysfunction might prevent the establishment of these pathological cycles. However, once established, successful intervention may require simultaneous targeting of the primary lesion and points in the downstream injury cascade [185]. This principle is demonstrated in stroke recovery, where combination therapies targeting inflammation, oxidative stress, and vascular function show greater efficacy than single-target approaches [204]. Similarly, optimal therapeutic strategies may need combinatorial approaches that evolve in line with disease progression (e.g., drug resistance) [205].

Several additional factors may sway the severity of disease and increase resistance to targeted therapeutic approaches, including genetic background and modifier gene effects, variation in ion channel compensation, and environmental variables (e.g., diagnosis delays, illnesses, status events). Because ion channel genes are expressed early in development, alterations in channel function may shape brain structure and function even before birth when channel dysfunction cannot be treated clinically [206,207].

The need for timing-specific intervention strategies, development of targeted delivery methods, and design of multi-modal therapeutic approaches all represent crucial areas for further progress. Enhanced discovery of biomarkers informative for disease stage and treatment response will be essential for optimizing therapeutic interventions. The translation of these findings to clinical practice will require careful consideration of optimal intervention timing, combination therapy protocols, and methods for patient-specific treatment selection.

## Supplementary material

Online supplementary material 1

## Abbreviations

AAV9: AAV serotype 9
ADNFLE: autosomal dominant nocturnal frontal lobe epilepsy
AIS: Axon initial segment
AP: Action potential
ARB: angiotensin receptor blocker
ASD: autism spectrum disorder
ASMs: antiseizure medications
ASO: antisense oligonucleotide
BBB: blood–brain barrier
BFNIS: familial neonatal-infantile seizures
CRISPR: Clustered Regularly Interspaced Short Palindromic Repeats
DEE: developmental and epileptic encephalopathies
DS: Dravet syndrome
EIMFS: malignant migrating focal seizures of infancy
ETC: Electron transport chain
GEFS+: Genetic Epilepsy with Febrile Seizures Plus
Gabra2: alpha2 subunit of the aminobutyric acid type A receptor
GoF: gain-of-function
ICV: intracerebroventricular
ID: intellectual disability
I_Na_: peak current
I_NaP_: persistent sodium current
I_NaR_: resurgent current
Kv: potassium channels
LoF: loss-of-function
MSNs: medium spiny neurons
NMDA: N-methyl-d-aspartate glutamate
NOR: Nodes of Ranvier
NREM: Non-Rapid-Eye-Movement
Nav: voltage-gated sodium channel
PV: parvalbumin
REM: Rapid-Eye-Movement
ROS: reactive oxygen species
SCN: suprachiasmatic nucleus
SO: stratum-oriens
SST: somatostatin
SUDEP: sudden unexplained death in epilepsy
SeL(F)IE: self-limited familial infantile epilepsy
TANGO: targeted augmentation of nuclear gene output
TBI: traumatic brain injury
VIP: vasoactive intestinal peptide
WT: wildtype
gtKO: gene-trap knockout
iPSC: induced pluripotent stem cell
mPFC: medial prefrontal cortex
shRNA: short hairpin RNA
