# Supplementary material for: Transgenic mouse models of sodium and potassium channelopathies in epilepsy: insights into disease mechanisms and therapeutics
Source: Biosci Rep. 2025 Oct 28;45(10):567–95. doi: 10.1042/BSR20253356 (PMC12784352; doi:10.1042/BSR20253356)
Supplement: Online supplementary material 1 [file bsr-45-10-BSR20253356-s001.docx]

**Table S1**. Key phosphorylation sites and physiological consequences in Nav and Kv channels.

| **Channel** | **Kinase** | **Site** | **Location in Channel** | **Physiological Consequence** | | **Citation** | |
| --- | --- | --- | --- | --- | --- | --- | --- |
| **Nav1.1** | PKA | S573 | DI-II linker | Reduced Na+ current | | (208) | |
|  | PKA | S610 | DI-II linker | Reduced Na+ current | | (208) | |
|  | PKA | S623 | DI-II linker | Reduced Na+ current | | (208) | |
| **Nav1.2** | PKA | S573 | DI-II linker | Reduced peak Na+ current, dopamine-dependent modulation | | (182) | |
|  | PKA | S610 | DI-II linker | Reduced Na+ current | | (209) | |
|  | PKA | S623 | DI-II linker | Reduced Na+ current | | (209) | |
|  | PKA | S687 | DI-II linker | Reduced Na+ current | | (209) | |
|  | PKC | S554 | DI-II linker | Channel modulation | | (208) | |
|  | PKC | S576 | DI-II linker | Channel modulation | | (208) | |
|  | PKC | S1506 | DIII-IV linker | Slower inactivation | | (208) | |
|  | CK2 | S1112 | DII-III linker | Modulates ankyrin-G interaction | | (208) | |
|  | CK2 | S1124 | DII-III linker | Modulates ankyrin-G interaction | | (208) | |
|  | CK2 | S1126 | DII-III linker | Modulates ankyrin-G interaction | | (208) | |
| **Nav1.6** | CaMKII | S561 | DI-II linker | Enhanced transient and persistent currents | (183) | |  |
|  | CaMKII | S641/T642 | DI-II linker | Enhanced transient and persistent currents, 5.8 mV hyperpolarizing shift in activation | (183) | |  |
|  | p38 MAPK | S553 | DI-II linker | Decreased current via Nedd4-mediated endocytosis, creates Pro-Gly-Ser-Pro motif | (18) | |  |
|  | PKA/PKC | Various | DI-II linker | Modest current reduction (less than Nav1.2) | (18) | |  |
| **Kv1.2** | PKA | S449 | C-terminus | Increased channel activity, enhanced surface expression | | (210) | |
|  | Unknown | S440 | C-terminus | Surface expression regulation | | (211) | |
|  | Unknown | S441 | C-terminus | Surface expression regulation | | (211) | |
| **Kv2.1** | CDK5 | Multiple | C-terminus | Constitutive phosphorylation maintenance | | (17) | |
|  | Calcineurin | S603 | C-terminus | Activity-dependent dephosphorylation, hyperpolarizing shift in activation | | (212) | |
|  | p38 MAPK | S800 | C-terminus | Pro-apoptotic surface expression increase | | (213) | |
|  | Src | Y124 | N-terminus | Pro-apoptotic modulation | | (213) | |
|  | Unknown | S567 | C-terminus | Hyperpolarizing shift in voltage-dependent gating | | (212) | |
|  | Unknown | S655 | C-terminus | Hyperpolarizing shift in voltage-dependent gating | | (212) | |
| **Kv4.2** | PKA | S552 | C-terminus | Depolarizing shift in activation (requires KChIP3), internalization from dendritic spines | | (214) | |
|  | CaMKII | S438 | C-terminus | Increased protein levels, no biophysical changes | | (215) | |
|  | CaMKII | S459 | C-terminus | Increased protein levels, no biophysical changes | | (215) | |
|  | PKC/PKA | General | C-terminus | Depolarizing shift in activation curve | | (216) | |
| **Kv7.2** | PKA | S52 | N-terminus | Enhanced KCNQ2 currents | | (217) | |
|  | CDK5 | S427 | C-terminus (PIP2 domain) | Maintains low PIP2 affinity for GPCR regulation | | (218) | |
|  | p38 MAPK | S436 | C-terminus (PIP2 domain) | Maintains low PIP2 affinity for GPCR regulation | | (218) | |
|  | PKA | S438 | C-terminus (PIP2 domain) | Maintains low PIP2 affinity for GPCR regulation | | (218) | |
|  | CaMKII | S446 | C-terminus (PIP2 domain) | Maintains low PIP2 affinity for GPCR regulation | | (218) | |
|  | PKA | S455 | C-terminus (PIP2 domain) | Maintains low PIP2 affinity for GPCR regulation | | (218) | |
| **Kv7.3** | Unknown | S579 | C-terminus | Essential for functional expression | | (219) | |
|  | Unknown | T580 | C-terminus | Essential for functional expression | | (219) | |

**References**

**208.** Johnson, R.P., El-Yazbi, A.F., Hughes, M.F., Schriemer, D.C., Walsh, E.J., Walsh, M.P. et al. (2009) Identification and Functional Characterization of Protein Kinase A-catalyzed Phosphorylation of Potassium Channel Kv1.2 at Serine 449. Journal of Biological Chemistry 284, 16562–16574 https://doi.org/10.1074/jbc.M109.010918

**209.** Yang, J.W., Vacher, H., Park, K.S., Clark, E. and Trimmer, JS. (2007) Trafficking-dependent phosphorylation of Kv1.2 regulates voltage-gated potassium channel cell surface expression. Proc. Natl. Acad. Sci. U.S.A 104, 20055–20060 https://doi.org/10.1073/pnas.0708574104

**210.** Park, K.S., Mohapatra, D.P., Misonou, H. and Trimmer, JS. (2006) Graded Regulation of the Kv2.1 Potassium Channel by Variable Phosphorylation. Science 313, 976–979 https://doi.org/10.1126/science.1124254

**211.** Redman, P.T., He, K., Hartnett, K.A., Jefferson, B.S., Hu, L., Rosenberg, P.A. et al. (2007) Apoptotic surge of potassium currents is mediated by p38 phosphorylation of Kv2.1. Proc. Natl. Acad. Sci. U.S.A. 104, 3568–3573 https://doi.org/10.1073/pnas.0610159104, 17360683

**212.** Schrader, L.A., Anderson, A.E., Mayne, A., Pfaffinger, P.J. and Sweatt, JD. (2002) PKA modulation of Kv4.2-encoded A-type potassium channels requires formation of a supramolecular complex. J. Neurosci. 22, 10123–10133 https://doi.org/10.1523/JNEUROSCI.22-23-10123.2002, 12451113

**213.** Varga, A.W., Yuan, L.-L., Anderson, A.E., Schrader, L.A., Wu, G.-Y., Gatchel, J.R. et al. (2004) Calcium–calmodulin-dependent kinase ii modulates kv4.2 channel expression and upregulates neuronal a-type potassium cualcium–Calmodulin-Dependent Kinase II Modulates Kv4.2 Channel Expression and Upregulates Neuronal A-Type Potassium Currents. J. Neurosci. 24, 3643–3654 https://doi.org/10.1523/JNEUROSCI.0154-04.2004

**214.** Hoffman, D.A. and Johnston, D. (1998) Downregulation of transient K+ channels in dendrites of hippocampal CA1 pyramidal neurons by activation of PKA and PKC. J. Neurosci. 18, 3521–3528 https://doi.org/10.1523/JNEUROSCI.18-10-03521.1998, 9570783

**215.** Schroeder, B.C., Kubisch, C., Stein, V. and Jentsch, TJ. (1998) Moderate loss of function of cyclic-AMP-modulated KCNQ2/KCNQ3 K+ channels causes epilepsy. Nature 396, 687–690 https://doi.org/10.1038/25367

**216.** Salzer, I., Erdem, F.A., Chen, W.-Q., Heo, S., Koenig, X., Schicker, K.W. et al. (2017) Phosphorylation regulates the sensitivity of voltage-gated Kv7.2 channels towards phosphatidylinositol-4,5-bisphosphate. J. Physiol. (Lond.) 595, 759–776 https://doi.org/10.1113/JP273274, 27621207

**217.** Surti, T.S., Huang, L., Jan, Y.N., Jan, L.Y. and Cooper, EC. (2005) Identification by mass spectrometry and functional characterization of two phosphorylation sites of KCNQ2/KCNQ3 channels. Proc. Natl. Acad. Sci. U.S.A. 102, 17828–17833 https://doi.org/10.1073/pnas.0509122102, 16319223

**218.** Martin, M.S., Dutt, K., Papale, L.A., Dubé, C.M., Dutton, S.B., et al and Haan, G. de. (2010) Altered function of the SCN1A voltage-gated sodium channel leads to gamma-aminobutyric acid-ergic (GABAergic) interneuron abnormalities. J. Biol. Chem. 285, 9823–9834, S0021-9258(19)55038-4 https://doi.org/10.1074/jbc.M109.078568, 20100831

**219.** Burgess, D.L., Kohrman, D.C., Galt, J., Plummer, N.W., Jones, J.M., Spear, B, et al. (1995) Mutation of a new sodium channel gene, Scn8a, in the mouse mutant “motor endplate disease”. Nat. Genet. 10, 461–465 https://doi.org/10.1038/ng0895-461, 7670495
